# Conjugate heat transfer study of various cooling structures and sensitivity analysis of overall cooling effectiveness

**DOI:** 10.1038/s41598-022-23948-6

**Published:** 2022-11-10

**Authors:** Runzhou Liu, Haiwang Li, Ruquan You, Yi Huang, Zhi Tao

**Affiliations:** 1 Research Institute of Aero-Engine, Beihang University, 37 Xueyuan Rd, Haidian District, Beijing, 100191 China; 2 School of Energy and Power Engineering, Beihang University, 37 Xueyuan Rd, Haidian District, Beijing, 100191 China; 3 National Key Laboratory of Science and Technology on Aero Engines Aero-Thermodynamics, Beihang University, 37 Xueyuan Rd, Haidian District, Beijing, 100191 China

**Keywords:** Engineering, Aerospace engineering, Mechanical engineering

## Abstract

The conjugate heat transfer of a turbine blade is influenced by several factors. To analyze the influence of each factor, the published one-dimensional conjugate heat transfer model was improved through theoretical analysis in this study. An overall cooling effectiveness equation containing three dimensionless parameters (adiabatic film cooling effectiveness *η*, Biot number on the mainstream side *Bi*_*g*_, and ratio between the heat transfer coefficients of the external and internal walls *h*_*g*_/*h*_*i*_) was obtained. The sensitivity of the overall cooling effectiveness *ϕ* to these three parameters was obtained through a multi-parameter sensitivity analysis. The results showed that increasing *η* could improve *ϕ* the most effectively. The interactions between the dimensionless parameters were analyzed by developing sensitivity charts. The results showed that increasing *η* from 0.4 to 0.5 could reduce the sensitivity of *ϕ* to the other two parameters by approximately 15%, whereas increasing *Bi*_*g*_ had little effect on the sensitivity of *ϕ* to each dimensionless parameter. Increasing *h*_*g*_/*h*_*i*_ could improve the sensitivity to *η*. The above conclusions could also be applied to the plate film hole and plate impingement effusion structures. The effects of different internal cooling structures and film hole structures on the three dimensionless parameters were studied by performing numerical simulations, which verified the accuracy of the one-dimensional conjugate heat transfer model in this study. The results showed that the internal cooling structures had little effect on the distribution of *η* and *Bi*_*g*_. The heat transfer coefficient on the coolant side could be effectively improved by installing film holes. The film hole structures mainly affected *ϕ* by influencing the distribution of *η*.

## Introduction

The thrust demand of modern aero-engines is increasing. According to the mechanism of gas turbines, an important method of improving thrust is increasing the turbine inlet temperature. However, a higher turbine inlet temperature presents a significant challenge for turbine blade design. The required turbine inlet temperature increase cannot be achieved simply by increasing the temperature tolerances of the materials. Therefore, efficient turbine blade cooling systems have become of great interest in the field of turbine blade heat transfer.

At present, turbine blade cooling systems are mainly divided into internal and external cooling systems. To evaluate external film cooling, the adiabatic film cooling effectiveness *η* is given by

1 $$\eta = \frac{{T_{g} - T_{aw} }}{{T_{g} - T_{c} }}.$$

Studies on film cooling have mainly been focused on film hole structures, the aerodynamic parameters of the mainstream and coolant, etc. Zeng et al.^1^ investigated the influence of the density ratio on *η* by performing a numerical simulation. Gritsch et al.^2,3^ examined flat film hole structures, and their results showed that the mainstream Mach number had little influence on the film cooling effectiveness. Ammari et al.^4^ showed that the density ratio of the coolant and mainstream had obvious effects on the heat transfer coefficient. Drost et al.^5^ studied the influence of the mainstream Reynolds number *Re*_*g*_ and turbulence intensity on film cooling using transient liquid crystals. With the development of film-cooling technology, shaped holes have gradually been applied in turbine blade design. Schulz et al.^6–8^ studied the film cooling effectiveness of cylindrical holes, fan-shaped holes, laidback fan-shaped holes, and laidback holes under different mainstream Mach numbers and turbulence intensity by conducting experiments and numerical simulations.

Impingement jets, serpentine passages with ribs, and pin-fins are the most commonly used internal cooling structures^9^. Impingement jets constitute the most effective means of enhancing heat transfer; however, the complexity of the structure increases the processing difficulty and weakens the strength. Therefore, impingement jets are generally used to cool the leading edges of guide vanes. To improve the heat transfer coefficient, a rib turbulator is usually arranged in a serpentine passage. Rib-fins are typically applied at the trailing edge to improve the strength and heat transfer. Han et al.^10–13^ performed detailed studies of typical impingement jets, rib turbulators, dimples, and novel internal cooling structures. In 2014, Wright et al.^14^ summarized the internal cooling results of the past decade and introduced new cooling design concepts.

The adiabatic film cooling effectiveness can be used to evaluate the external film cooling, and the Nusselt number and pressure-loss coefficient can be employed to evaluate the internal cooling structure. Although the above parameters can be utilized to evaluate film cooling and internal cooling, they cannot reflect the interaction between the two cooling systems or the conjugate heat transfer. The temperature of the blade surface is an important concern in turbine blade cooling systems and a direct reflection of conjugate heat transfer. The overall cooling effectiveness *ϕ* is a dimensionless parameter that represents the blade surface temperature, which can provide a direct reference for engineers. *ϕ* is defined as follows:

2 $$\phi = \frac{{T_{g} - T_{w} }}{{T_{g} - T_{c} }}.$$

The overall cooling effectiveness considers the coupled effects of external film cooling, solid heat conduction, and internal cooling, which is of great significance in the study of turbine blade heat transfer.

Bohn et al.^15,16^ studied the overall cooling effectiveness of a flat-film hole model by performing a numerical simulation. The maximum turbine inlet temperature could reach 2000 K under actual engine conditions; therefore, it is difficult to conduct actual engine experiments under laboratory conditions. Hence, matching the experimental results obtained under laboratory conditions with those acquired under actual engine conditions is the focus of current studies. Bogard et al.^17–26^ made a significant contribution to the matching principle of *ϕ*. The authors proposed a theory of dimensionless parameters that influence *ϕ* by establishing a one-dimensional conjugate heat transfer model. The results showed that the Biot number *Bi*_*g*_, heat transfer coefficient ratio *h*_*g*_/*h*_*i*_, adiabatic film cooling effectiveness *η*, and internal coolant warming factor ($\frac{{T}_{g}-{T}_{w,i}}{{T}_{g}-{T}_{c}}$) all affected the overall cooling effectiveness. Bogard et al.^19^ also experimentally studied the overall cooling effectiveness of the leading-edge region, and discussed how to match *Bi*_*g*_ and the heat transfer coefficient. Meanwhile, Xie et al.^27–29^ obtained the matching principle of overall cooling effectiveness under different conditions by analyzing typical turbine blade cooling structures and verified it through numerical simulations and experiments. The results showed that matching the temperature ratio and thermal conductivity was the most accurate method of obtaining experimental results under low-temperature conditions when the Reynolds numbers of the mainstream and coolant were similar. Chavez et al.^20,30^ studied the effects of the mainstream flow angle and arrangement of film holes on *ϕ* after matching *Bi*_*g*_. A one-dimensional conjugated heat transfer model was used to predict *ϕ* accurately.

Thus, many scholars have investigated the conjugate heat transfer of turbine blades. Their research has mainly involved the matching principle between laboratory and actual engine conditions and the effects of different cooling structures on *ϕ*. However, most studies have only investigated the effects of geometric and aerodynamic parameters on *ϕ* after matching *Bi*_*g*_ or temperature ratio. The aerodynamic and geometric parameters are very complex and depend on the actual conditions of the turbine blades. After changing one parameter, *ϕ* also changes. Therefore, the coupled mechanism of film cooling, solid heat conduction, and internal cooling on *ϕ* cannot be obtained simply by studying the effects of the aerodynamic or geometric parameters. In this study, *ϕ* was simplified to an equation containing three dimensionless parameters by improving the published one-dimensional conjugate heat transfer model. These three dimensionless parameters are the adiabatic film cooling effectiveness (*η*), Biot number on the mainstream side (*Bi*_*g*_), and heat transfer coefficient ratio (*h*_*g*_/*h*_*i*_), which are related to the film cooling, solid heat conduction, and internal cooling, respectively. Sensitivity analysis was conducted to obtain the coupled effects of these three parameters on *ϕ*. Subsequently, the effects of different aerodynamic parameters and cooling structures on the three dimensionless parameters were studied by numerical simulation. Finally, the accuracy of the improved one-dimensional conjugate heat transfer model was verified through numerical simulation. In this study, three dimensionless parameters are constructed between the overall cooling effectiveness and aerodynamic/geometric parameters, which makes the investigation of conjugate heat transfer more systematic. At the same time, this study also verifies the accuracy of one-dimensional conjugate heat transfer model. The results show that the model and sensitivity charts can play an auxiliary role in the early stage of turbine blade design.

## Analogy theory analysis

### One-dimensional conjugate heat transfer model

**Figure 1:**
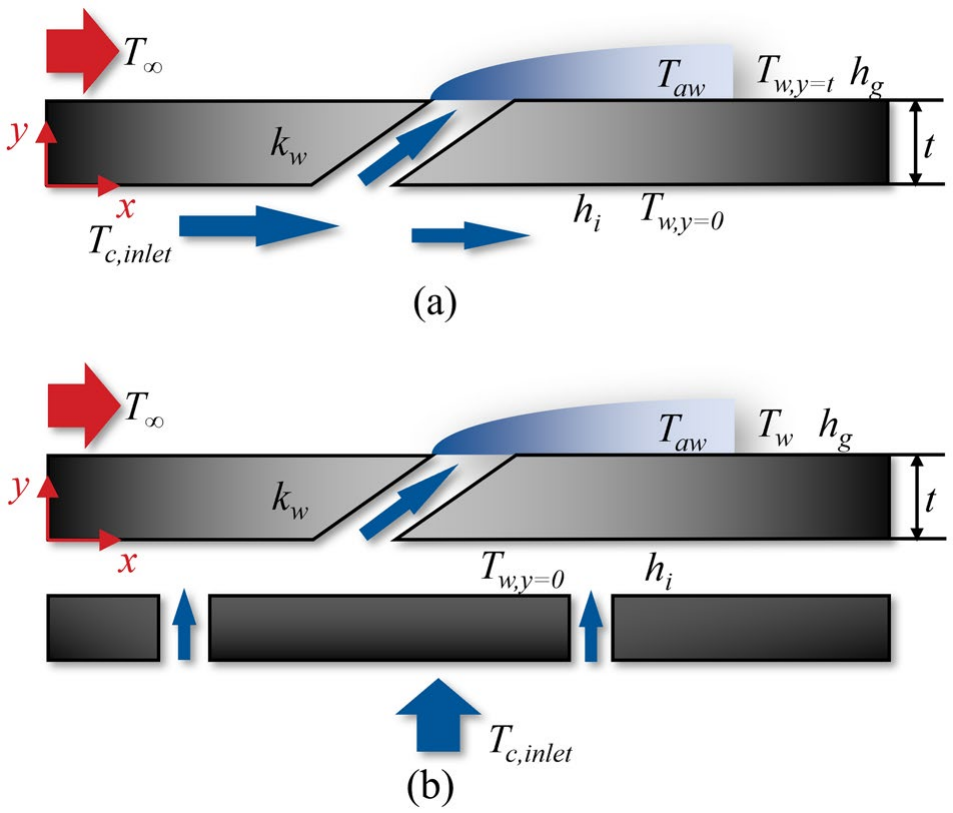
Schematic diagrams of cooling plate. (**a**) Film hole cooling plate; (**b**) Impingement effusion cooling plate.

Film cooling and impingement effusion are two common cooling methods used in current turbine blades. Therefore, a film cooling plate and an impingement effusion plate were used as the analysis objects to establish a one-dimensional conjugate heat transfer model, as shown in Fig. 1. Bogard et al.^19^ made outstanding contributions to a one-dimensional conjugate heat transfer model and defined *η* as follows:

3 $$\eta_{standard} = \frac{{T_{g} - T_{aw} }}{{T_{g} - T_{c,exit} }},$$

where *T*_*c,exit*_ denotes the coolant temperature at the outlet of the film hole. Evidently, *T*_*c,exit*_ cannot can be directly applied in turbine blade design. First, *T*_*c,exit*_ changes with the geometry of cooling structures. Adopting this parameter in the definition of *η* will negate the significance of modelling overall cooling effectiveness. Second, it was difficult to obtain the coolant temperature at the outlet of the film hole accurately. In contrast, *T*_*c,inlet*_ (coolant temperature at the inlet of the cooling structure) is commonly used in turbine blade design and does not change with variations in the cooling structure. Therefore, *η* used in this study is defined as follows:

4 $$\eta = \frac{{T_{g} - T_{aw} }}{{T_{g} - T_{c,inlet} }}.$$

The boundary condition of the third kind is

5 $$- \lambda \left( {\frac{\partial T}{{\partial n}}} \right)_{w} = h\left( {T_{w} - T_{f} } \right),$$

where *n* is the outer normal direction of the heat transfer surface, *w* is the surface of the solid material, and *f* is the fluid. In this study, the heat transfer coefficient on the mainstream side *h*_*g*_ is defined as follows:

6 $$q_{g} = h_{g} \left( {T_{aw} - T_{w,y = t} } \right),$$

where *T*_*aw*_ denotes the adiabatic wall temperature with film hole cooling. The heat transfer coefficient on the coolant side (*h*_*i*_) is defined as

7 $$q_{i} = h_{i} \left( {T_{w,y = 0} - T_{c,inlet} } \right)$$

According to the boundary conditions of the third type, the boundary conditions of the model in Fig. 1 are as follows:

8 $$\left\{ {\begin{array}{*{20}l} {k\frac{\partial T}{{\partial y}}|_{y = 0} = h_{i} \left( {T|_{y = 0} - T_{c,inlet} } \right)} \hfill \\ { - k\frac{\partial T}{{\partial y}}|_{y = t} = h_{g} \left( {T|_{y = t} - T_{aw} } \right)} \hfill \\ \end{array} } \right..$$

To simplify Eq. (8), the distribution of the dimensionless temperature can be expressed as

9 $$\theta = \frac{{T_{\infty } - T}}{{T_{\infty } - T_{c,inlet} }}.$$

In addition, the dimensionless distance, *Y* = *y*/*t*, was defined. By substituting these dimensionless parameters into Eq. (8), and combining it with Eq. (4), we obtain

10 $$\left\{ {\begin{array}{*{20}l} {\frac{\partial \theta }{{\partial Y}}|_{Y = 0} = \frac{{h_{i} }}{{h_{g} }}Bi_{g} \left( {\theta |_{Y = 0} - 1} \right)} \hfill \\ {\frac{\partial \theta }{{\partial Y}}|_{Y = 1} = Bi_{g} \left( {\eta - \theta |_{Y = 1} } \right)} \hfill \\ \end{array} } \right..$$

The governing equation for the two-dimensional steady state without an internal heat source is given by

11 $$\frac{{\partial^{2} T}}{{\partial x^{2} }} + \frac{{\partial^{2} T}}{{\partial y^{2} }} = 0.$$

In this study, the film hole plate and impingement effusion plate were simplified as one-dimensional heat transfer models. Therefore, the governing equation is

12 $$\frac{{\partial^{2} T}}{{\partial y^{2} }} = 0.$$

By substituting Eq. (9) into Eq. (12), we obtain

13 $$\frac{{\partial^{2} \theta }}{{\partial Y^{2} }} = 0.$$

The following conclusion can be drawn from Eq. (13):

14 $$\frac{\partial \theta }{{\partial Y}}|_{Y = 0} = \frac{\partial \theta }{{\partial Y}}|_{Y = 1} = \theta |_{Y = 1} - \theta |_{Y = 0} .$$

By combining Eqs. (10) and (14), we obtain

15 $$\theta |_{Y = 1} = \eta + \frac{1 - \eta }{{1 + h_{g} /h_{i} + Bi_{g} }}.$$

When *Y* = 1, $$\theta |_{Y = 1}$$ is

16 $$\theta |_{Y = 1} = \frac{{T_{\infty } - T_{w} }}{{T_{\infty } - T_{c,inlet} }}.$$

According to Eqs. (2), (16) also represents the overall cooling effectiveness *ϕ*. Therefore, the equation relating overall cooling effectiveness *ϕ* to the three aforementioned dimensionless parameters (adiabatic film cooling effectiveness *η*, heat transfer coefficient ratio *h*_*g*_/*h*_*i*_, and Biot number *Bi*_*g*_) under a one-dimensional steady state without an internal heat source is given by

17 $$\phi = \eta + \frac{1 - \eta }{{1 + h_{g} /h_{i} + Bi_{g} }}.$$

It can be observed from Eq. (17) that the overall cooling effectiveness is affected by three dimensionless parameters, which also indicates that the temperature of the blade surface is affected by the coupling effect of film cooling, solid heat conduction, and internal cooling. Compared with the previous study^17^, after changing the definitions of internal heat transfer coefficient (*h*_*i*_) and adiabatic film cooling effectiveness (*η*), the coolant warming factor *χ*, which represented the coolant warming level from the inlet to the outlet of the film hole, was excluded from the current equation. The definition of *χ* is $\chi =\left({T}_{\infty }-{T}_{c,exit}\right)/\left({T}_{\infty }-{T}_{c,inlet}\right)=\eta /{\eta }_{standard}$. Therefore, the effect represented by *χ* was included in the adiabatic film cooling effectiveness (*η*). This will make the relationships between the temperature of the blade surface and film cooling, solid heat conduction, and internal cooling clearer. The influence of each aspect will be corresponding to a dimensionless parameter (film cooling (*η*), solid heat conduction (*Bi*_*g*_) and internal cooling (*h*_*g*_/*h*_*i*_)).

### Sensitivity analysis of overall cooling effectiveness

Parameter sensitivity refers to the level at which the dependent variable changes with respect to the independent variable. If the dependent variable changes significantly, then the dependent variable has high sensitivity to and low robustness against this independent variable. In other words, when the dependent variable changes to a certain value, high sensitivity means that the independent variable needs to change less. To determine the effects of the independent variables on the dependent variable, partial derivatives can be performed. The partial derivative of *ϕ* with respect to each dimensionless parameter can be obtained by differentiating Eq. (17):

18 $$\frac{\partial \phi }{{\partial \eta }} = 1 - \frac{1}{{1 + h_{g} /h_{i} + Bi_{g} }}.$$

19 $$\frac{\partial \phi }{{\partial Bi_{g} }} = - \frac{1 - \eta }{{\left( {1 + h_{g} /h_{i} + Bi_{g} } \right)^{2} }}.$$

20 $$\frac{\partial \phi }{{\partial \left( {h_{g} /h_{i} } \right)}} = - \frac{1 - \eta }{{\left( {1 + h_{g} /h_{i} + Bi_{g} } \right)^{2} }}.$$

Based on the simulation results obtained in this study and published previously^21,27^, the representative values of each dimensionless parameter are listed in Table 1. According to Table 1 and Eqs. (18)–(20), we obtained the specific partial derivative results shown in Table 2. The partial derivative with respect to *η* is positive, indicating that it is positively correlated with *ϕ*. Meanwhile, the partial derivatives with respect to *Bi*_*g*_ and *h*_*g*_/*h*_*i*_ are negative, indicating that these two parameters are negatively correlated with *ϕ*. The larger the partial derivative, the more sensitive *ϕ* to this parameter. It can be observed that *ϕ* is the most sensitive to *η*, which indicates that improving *η* can most effectively improve *ϕ*.

**Table 1:** Values of three dimensionless parameters under engine working conditions.

Parameter	*η*	*Bi*_*g*_	*h*_*g*_/*h*_*i*_
Value	0.4	0.1	2

**Table 2:** Specific partial derivatives with respect to each dimensionless parameter.

Partial derivative	$\frac{\partial \phi }{{\partial \eta }}$	$\frac{\partial \phi }{{\partial Bi_{g} }}$	$\frac{\partial \phi }{{\partial \left( {h_{g} /h_{i} } \right)}}$
Value	0.677	− 0.062	− 0.062

**Figure 2:**
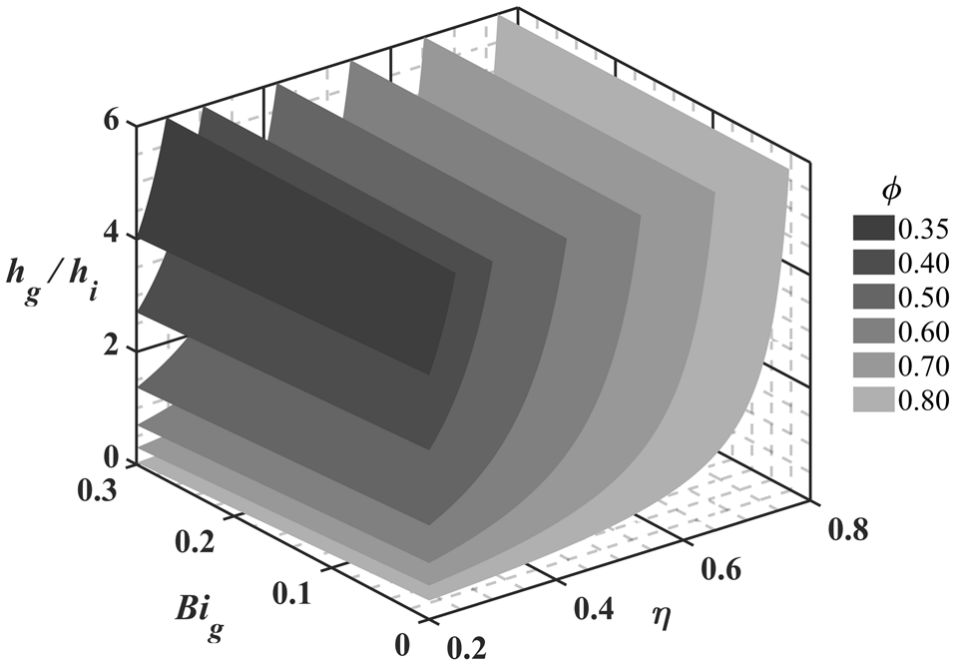
Isosurface of overall cooling effectiveness.

**Figure 3:**
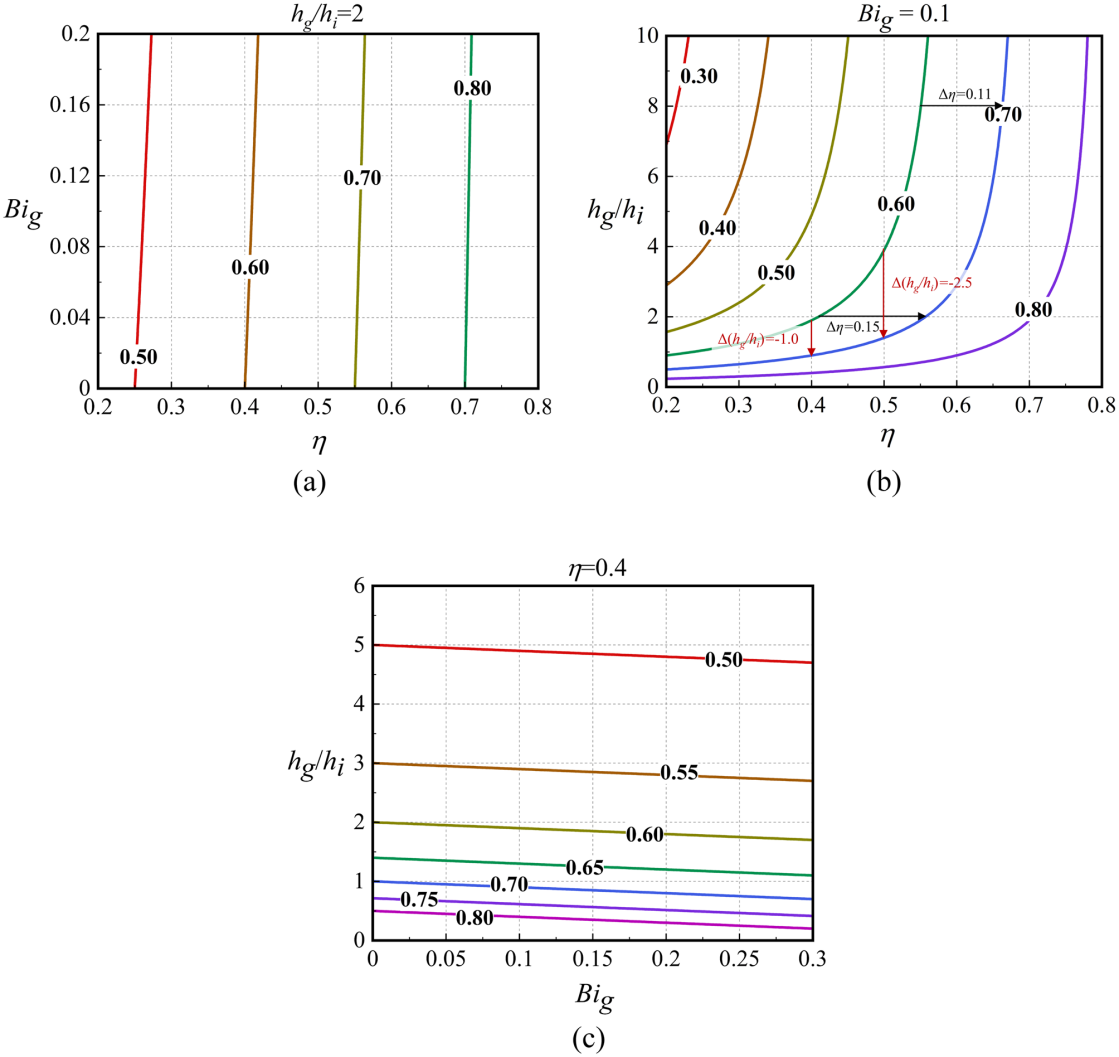
Contours of overall cooling effectiveness.

Through the above analysis, the relationship between *ϕ* and a single dimensionless parameter can be obtained. In addition, it is necessary to determine whether there are interactions between the three dimensionless parameters. Therefore, sensitivity charts were developed for this study, as shown in Fig. 2, which presents the isosurfaces of *ϕ* changes with three dimensionless parameters. When a dimensionless parameter remains constant, the extracted 2-D sensitivity charts (contours of *ϕ*) are shown in Fig. 3. As depicted in Fig. 3a, when *ϕ* increases by 0.1, regardless of *Bi*_*g*_, *η* must be increased by 0.15. In other words, the curves are parallel to one another, and the intervals between curves are equal. Thus, *η* does not affect the sensitivity of *ϕ* to itself. As shown by the black arrow in Fig. 3b, if *ϕ* increases from 0.6 to 0.7, then *η* needs to be increased from 0.42 to 0.57 when *h*_*g*_/*h*_*i*_ is 2. When *h*_*g*_/*h*_*i*_ increases to 8, *η* must be increased from 0.55 to 0.66. The adiabatic film cooling effectiveness must be increased by 0.15 and 0.11, respectively. Thus, the sensitivity of *ϕ* to *η* increases with increasing *h*_*g*_/*h*_*i*_. The slopes of all the curves in Fig. 3a are basically the same, indicating that *Bi*_*g*_ hardly affects the sensitivity of *ϕ* to *η*, which is because *Bi*_*g*_ is much less than *h*_*g*_/*h*_*i*_, and the effects on sensitivity can be ignored. Similarly, Fig. 3c demonstrates that *Bi*_*g*_ does not affect the sensitivity of *ϕ* to itself or to *h*_*g*_/*h*_*i*_. It can be seen from Fig. 3b that if *ϕ* increases from 0.6 to 0.7, *h*_*g*_/*h*_*i*_ needs to be reduced by 1.0 when *η* is 0.4. When *η* = 0.5, *h*_*g*_/*h*_*i*_ must be reduced by 2.5. Thus, an increase in *η* reduces the sensitivity of *ϕ* to *h*_*g*_/*h*_*i*_. The effects of *η* and *h*_*g*_/*h*_*i*_ on *Bi*_*g*_ are not evident in Fig. 3, but the conclusions can be obtained from Eqs. (18)–(20) and are summarized in Table 3.

**Table 3:** Multi-parameter sensitivity analysis results for overall cooling effectiveness.

	Sensitivity		
	*η*	*h*_*g*_/*h*_*i*_	*Bi*_*g*_
*η* (↑)	–	↓	↓
*h*_*g*_/*h*_*i*_ (↑)	↑	↓	↓
*Bi*_*g*_ (↑)	~	~	~

*↑* (*increase*) *↓*(*decrease*) –(*no effects*) ~ (*fewer effects*).

The above analysis demonstrates that *ϕ* can be improved by improving *η* and reducing *Bi*_*g*_ and *h*_*g*_/*h*_*i*_. To keep *ϕ* constant, *Bi*_*g*_ and *h*_*g*_/*h*_*i*_ need to be reduced further with the increase of *η*, which makes the design and process difficult. Therefore, these three dimensionless parameters must be properly selected to achieve the required overall cooling effectiveness. In the early stage of turbine blades design, engineers can refer to Figs. 2, 3 to select the appropriate dimensionless parameters. Next, the effects of the geometric and aerodynamic parameters on the three parameters were studied by numerical simulation. The accuracy of the one-dimensional conjugate heat transfer model was also verified.

## Numerical simulation method

### Numerical model and boundary conditions

**Figure 4:**
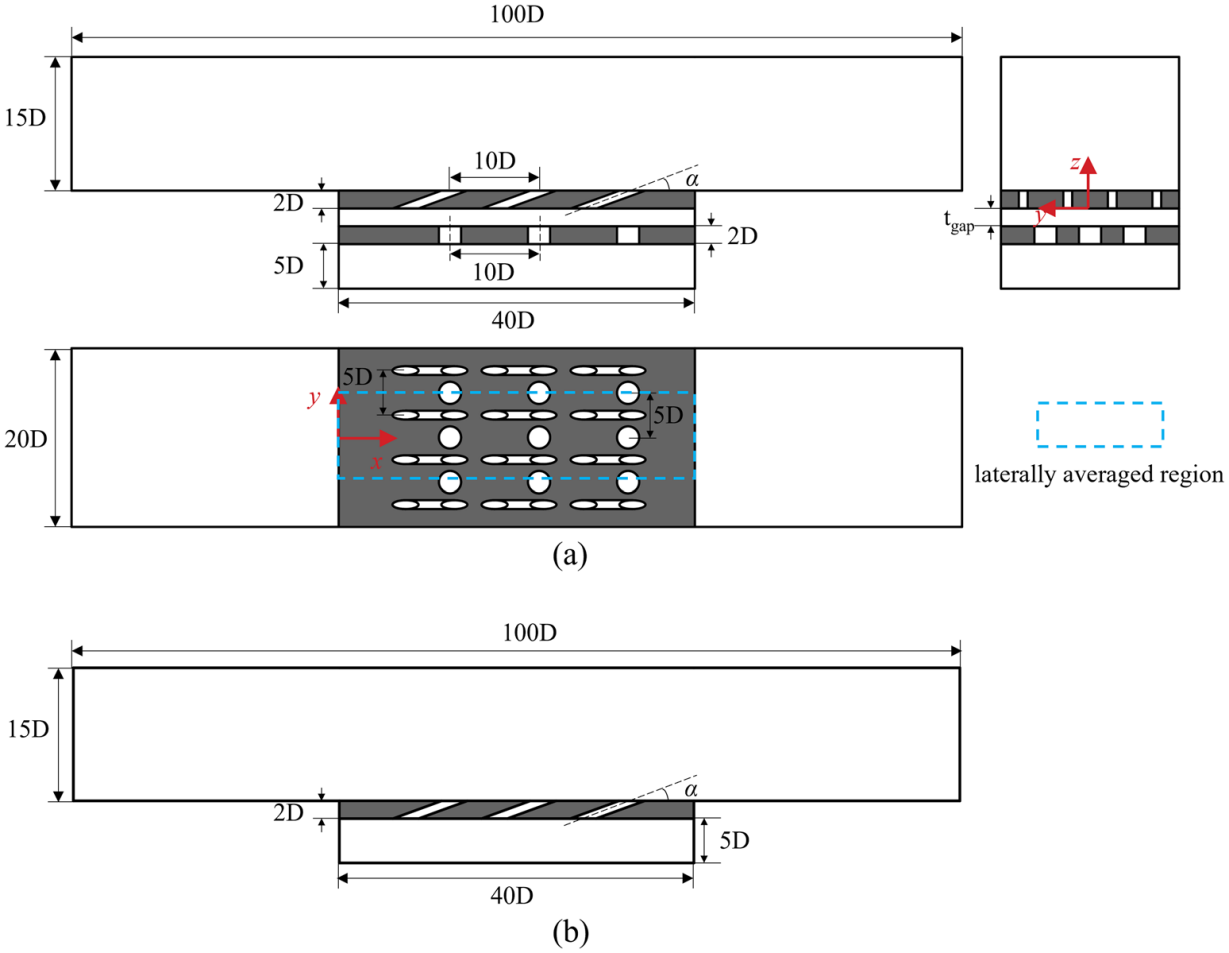
Geometric parameters of impingement-film hole model. (**a**) Impingement effusion model; (**b**) Film-hole model.

**Figure 5:**
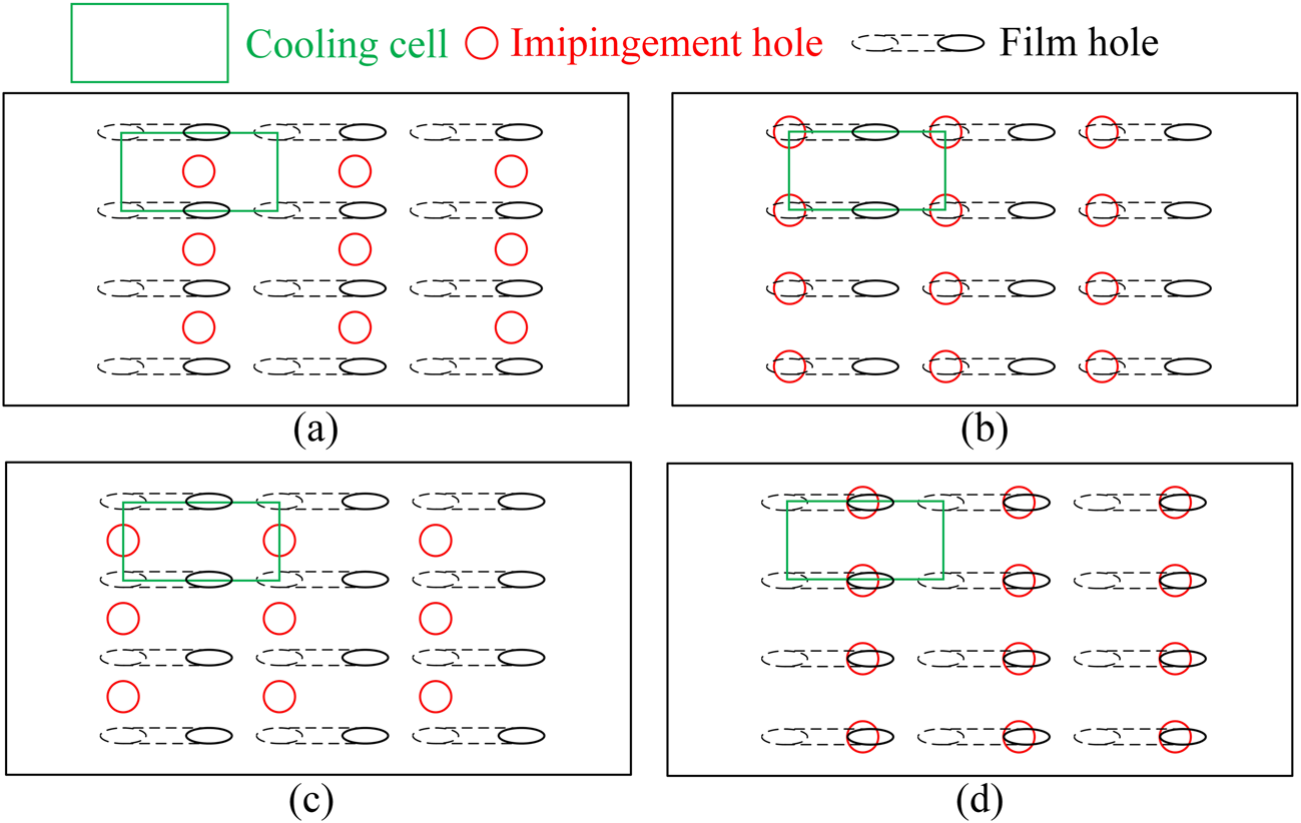
Layouts of impingement hole (**a**): staggered, (**b**): overlapped, (**c**): span parallel, (**d**): streamwise parallel).

**Figure 6:**
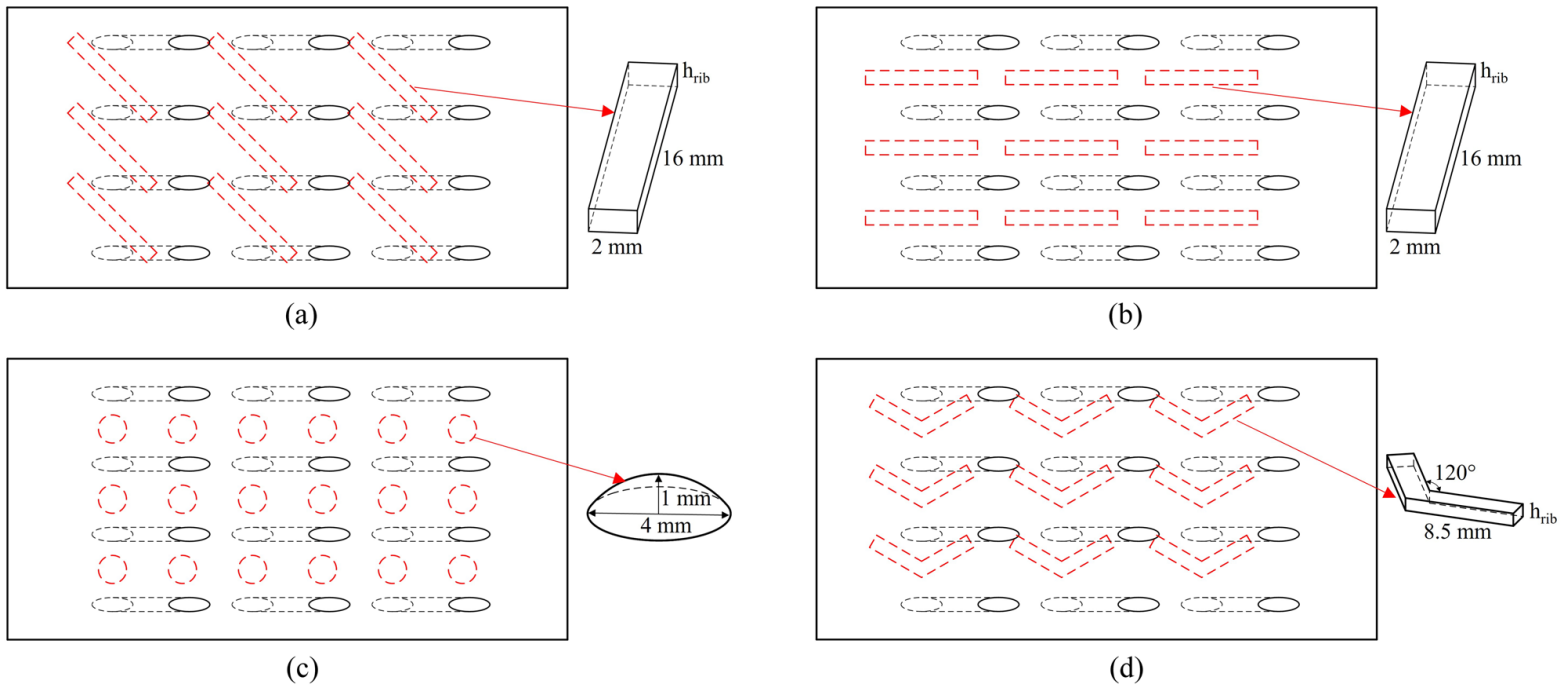
Schematic diagrams of rib turbulators. (**a**) 45°; (**b**) 90°; (**c**) Dimple; (**d**) V.

**Figure 7:**
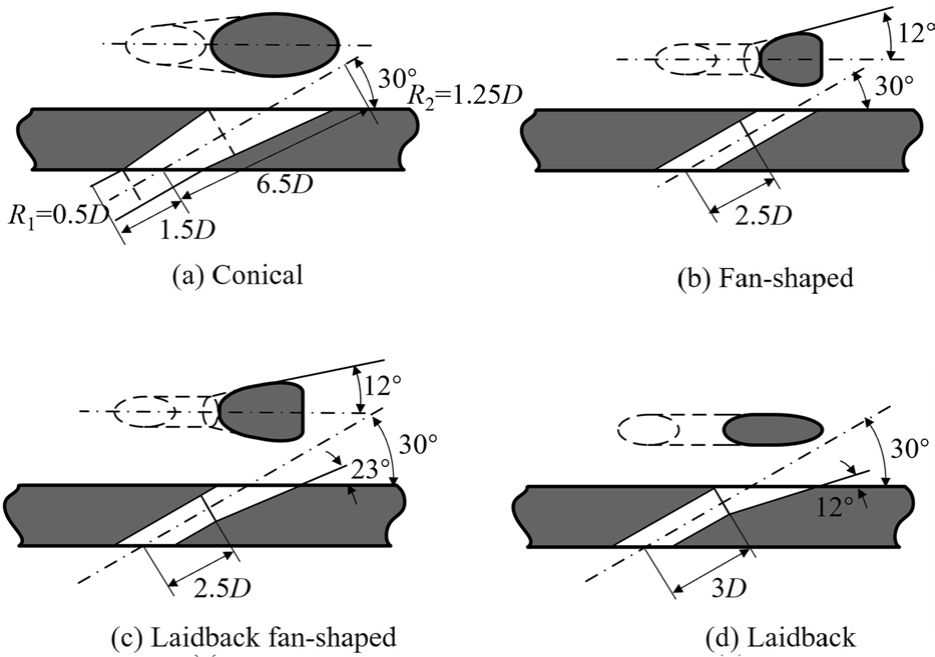
Typical shaped-hole in film cooling model.

In “One dimensional conjugate heat transfer model” section, the flat-plate cooling structures were studied to obtain the relationship between the improved one-dimensional conjugate heat transfer model and three dimensionless parameters. In order to obtain the influence of aerodynamic/geometry parameters on the three dimensionless parameters, this study will be carried out by numerical simulation. The simulation model adopted the flat-plate cooling structure matching with Fig. 1, as shown in Fig. 4. The characteristic length was the film hole diameter of the basic case (*D* = 2 mm). The geometry and other dimensionless parameters were based on it. To study the effects of film hole diameter *D*_*film*_, impingement hole diameter *D*_*imp*_, impingement hole layouts, impingement gap *t*_*gap*_, film hole structure, and film hole inclined angle *α*, these parameters were determined by classic published studies, as shown in Figs. 5, 6 and 7. There were four layouts of impingement holes, namely staggered, overlapped, span parallel and streamwise parallel, as shown in Fig. 5. Four different rib turbulators were studied. The layout and geometry of the ribs are shown in Fig. 6. Four typical shaped-holes were employed and the geometric parameters were shown in Fig. 7. The other geometric parameters are listed in Table 4.

**Table 4:** Numerical model geometric parameters.

Parameters	Values
*α*/°	20, 30
*D*_*film*_/mm	1.5, 2, 3
*D*_*imp*_/mm	3, 4, 5
*t*_*gap*_/mm	3, 4, 5, 6
*h*_*rib*_/mm	1

The all-numerical cases are listed in Table 5. Two cases (Ref-case #1: smooth internal channel without impingement flow; Ref-case #2: smooth internal channel with impingement flow) were selected as references. The left-most column of the Table 5 represented the variables of each case. For example, “gap distance” case was used to study the effects of impingement gap distance. Therefore, only the gap distance changed compared with Ref-case #2. The “rib turbulators” case represented that the model was equipped with four kinds of ribs shown in Fig. 6 on the basis of Ref-case #1. The “layouts of impingement hole” case represented the change of impingement hole layout based on the Ref-case #2, where Ref-case #2 case was the staggered layout. The case represented by “film/impingement hole diameter” was based on Ref-case #1/2, and the film/impingement hole diameter was changed. The “shaped-hole” case represented that the film hole structure and inclined angle were modified on the basis of Ref-case #1. In the following sections, the label represents the change of the simulation model based on these two reference cases, unless otherwise specified.

**Table 5:** Specific parameters for each case.

Case	Film hole diameter *D*_*film*_/mm	Film hole inclined angle *α*/°	Imp-hole diameter *D*_*imp*_/mm	Gap distance *t*_*gap*_/mm
Ref-case #1	2	20	–	–
Ref-case #2	2	20	4	4
Gap distance	2	20	4	4, 6, 10, 14, 20, 30
Rib turbulators	2	20	–	–
Layouts of impingement hole	2	20	4	4
Film hole diameter	1.5, 2, 2.5	20	–	–
Impingement hole diameter	2	20	3, 4, 5	4
Shaped-hole	2 (cylinder hole section)	30	–	–

The matching principle of *ϕ* between laboratory conditions and actual engine conditions has drawn the attention of many scholars. Liu et al.^27,28^ found that if *Re*_*g*_, the temperature ratio *T*_*g*_/*T*_*c*_, and blowing ratio *M* were matched, *ϕ* under laboratory conditions was consistent with that under actual engine conditions. Thus, the boundary conditions are listed in Table 6.

**Table 6:** Boundary conditions.

Parameter	Value(s)
*T*_*g*_/K	600
*T*_*c*_/K	303
*T*_*g*_/*T*_*c*_	1.98
*Re*_*g*_	2900, 3100, 3300, 3500, 3700
*M’*	1
*k*_*solid*_/(W/m·K)	10.6

**Figure 8:**
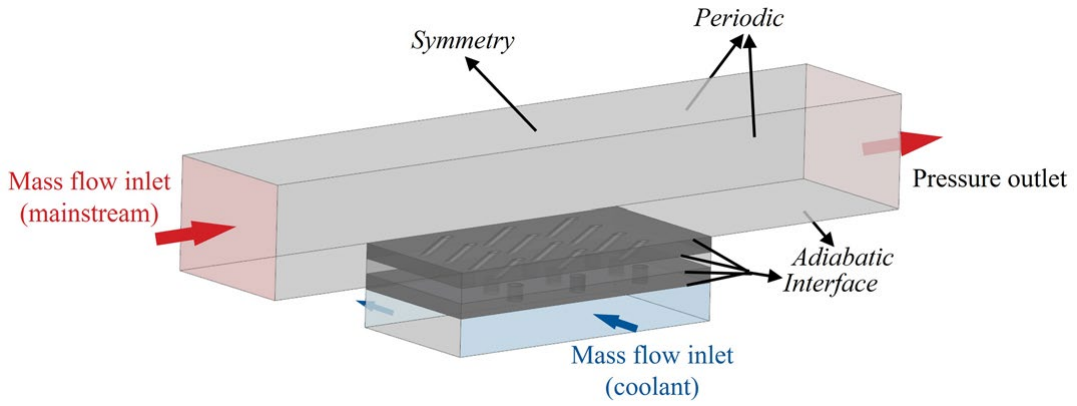
Boundary conditions of numerical model.

Figure 8 shows the specific boundary conditions of the numerical model. The mainstream and coolant inlets were employed to set the mass flow conditions, and the outlet was utilized to set the pressure boundary condition. To simulate the effects of cross-flow, coolant flowed in from the side (blue), and part of it flowed out from the other side. The other side was set as the mass flow outlet to ensure that the coolant flow rate satisfied the blowing ratio requirement. The mainstream Reynolds number was defined as ${Re}_{g}=\frac{{\rho }_{g}{u}_{g}D}{\mu }$. The flow rate of the coolant was determined using the equivalent blowing ratio *M'*. The standard blowing ratio was defined as $M=\frac{{\rho }_{c}{u}_{c}}{{\rho }_{g}{u}_{g}}$. In this study, *M'* was defined as $M^{\prime} = \frac{{\dot{m}_{c} /A_{c,all} }}{{\dot{m}_{g} /A_{g,inlet} }}$, where *A*_*c,all*_ was the sum of the outlet areas of all the film holes and *A*_*g,inlet*_ was the inlet area of the mainstream. The two sides of the mainstream channel were set as periodic boundary conditions. The coupled surfaces between the fluid and solid domains were set as the “Interface” with conservative heat flux in CFX 18.0. The other walls were set as adiabatic conditions. In the adiabatic simulation model, only the fluid domain was solved to obtain the adiabatic film cooling effectiveness *η*. Thus, all walls were adiabatic. The thermal conductivity of the blade was 20–25 W/m K under actual engine conditions^31^. In order to match *Bi*_*g*_ in this paper with the actual engine conditions, titanium alloy (10.6 W/m K) was selected as the solid material. The fluid domain was an ideal gas with dynamic viscosity set as the Sutherland formula. An empirical fitting correlation was adopted for the thermal conductivity *k* and specific heat capacity *Cp* of the solid and fluid.

### Numerical methods and reliability

**Figure 9:**
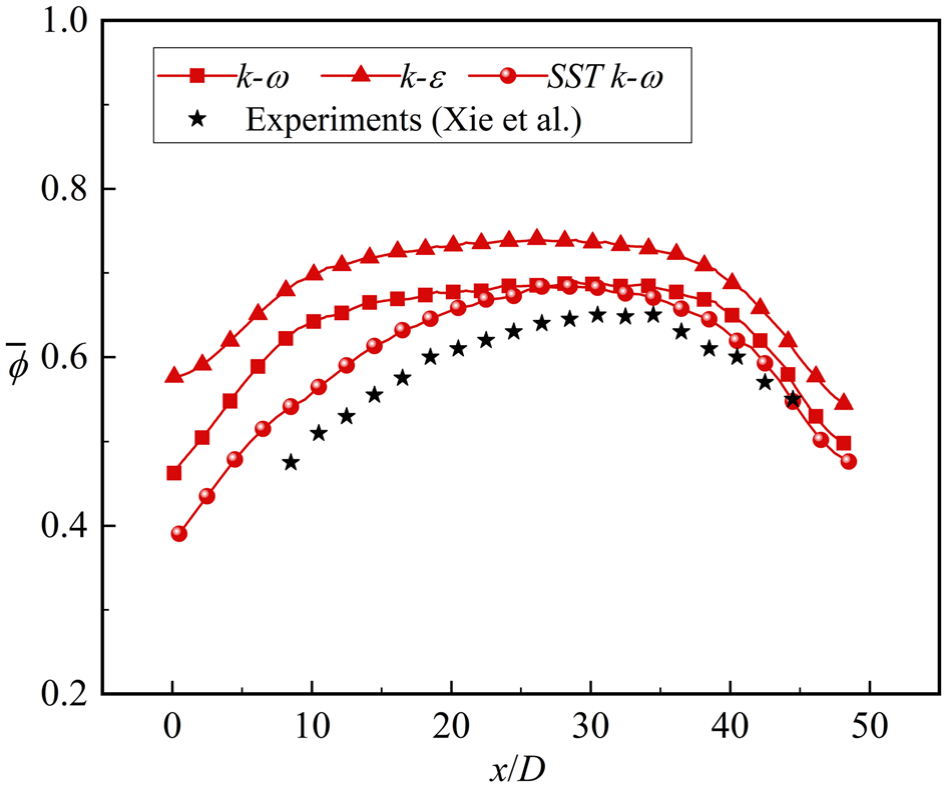
Validation of turbulence model.

The commercial software ICEM CFD and ANSYS CFX were employed to compute the unstructured mesh and solve the steady RANS equations, respectively. The meshes of the solid and fluid domains were separately computed. It can be observed from previously published reports^30,32–34^ that the SST *k–ω* model is suitable for various complex flows. Shi et al.^35^ studied the overall cooling effectiveness of an impingement effusion cooling structure through numerical simulation and experiments, using the SST *k–ω* and RNG *k–ε* turbulence models. The results showed that the overall cooling effectiveness obtained by the SST *k–ω* model was similar to the experimental results. In addition, this study combined the published literature^28^ to verify the turbulence models, as shown in Fig. 9. Therefore, the SST *k–ω* turbulence model was employed in this study. The inlet turbulence intensity was set as “Medium (Intensity = 5%)” in the CFX software.

**Figure 10:**
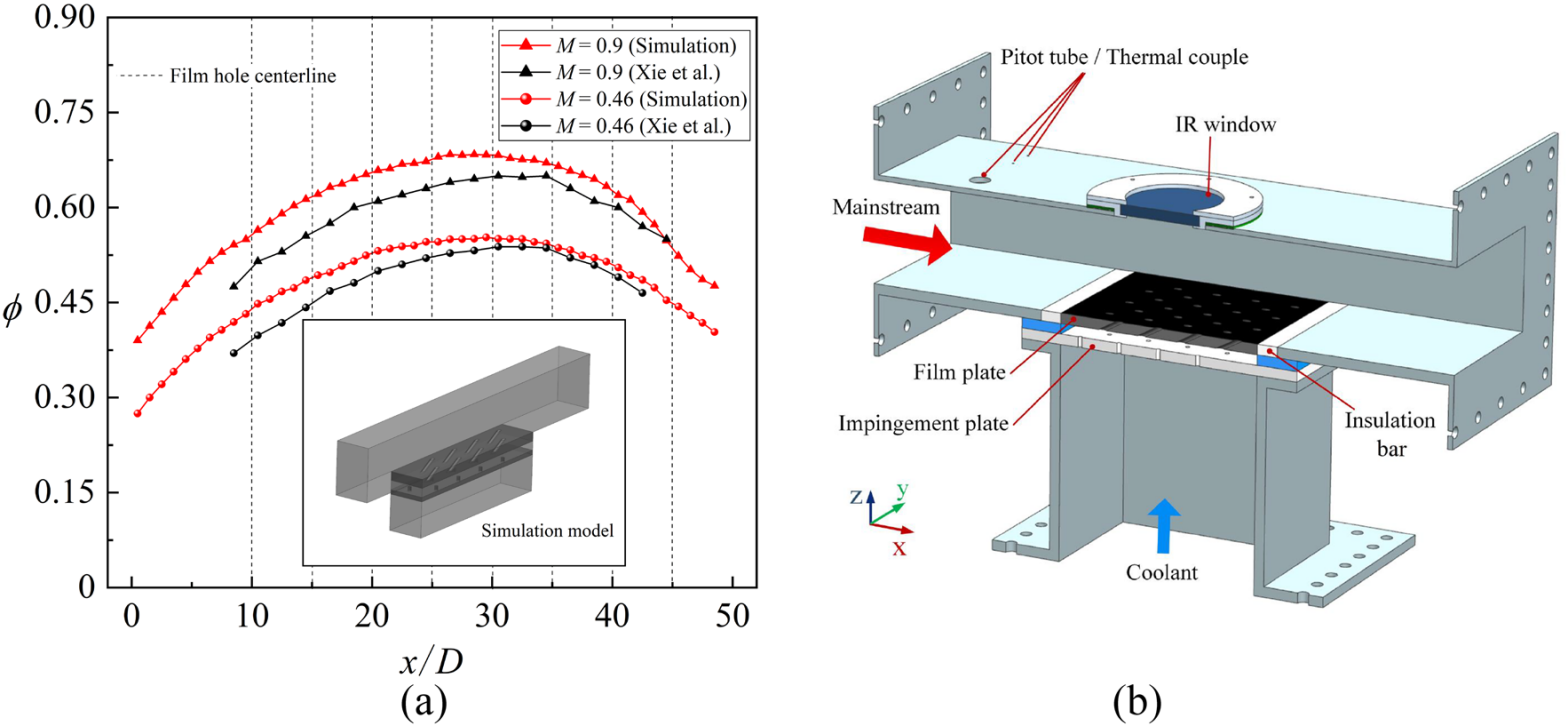
Comparison between numerical simulation and experimental results^28^. (**a**) Laterally averaged overall cooling effectiveness; (**b**) Schematic view of test Section^28^.

**Figure 11:**
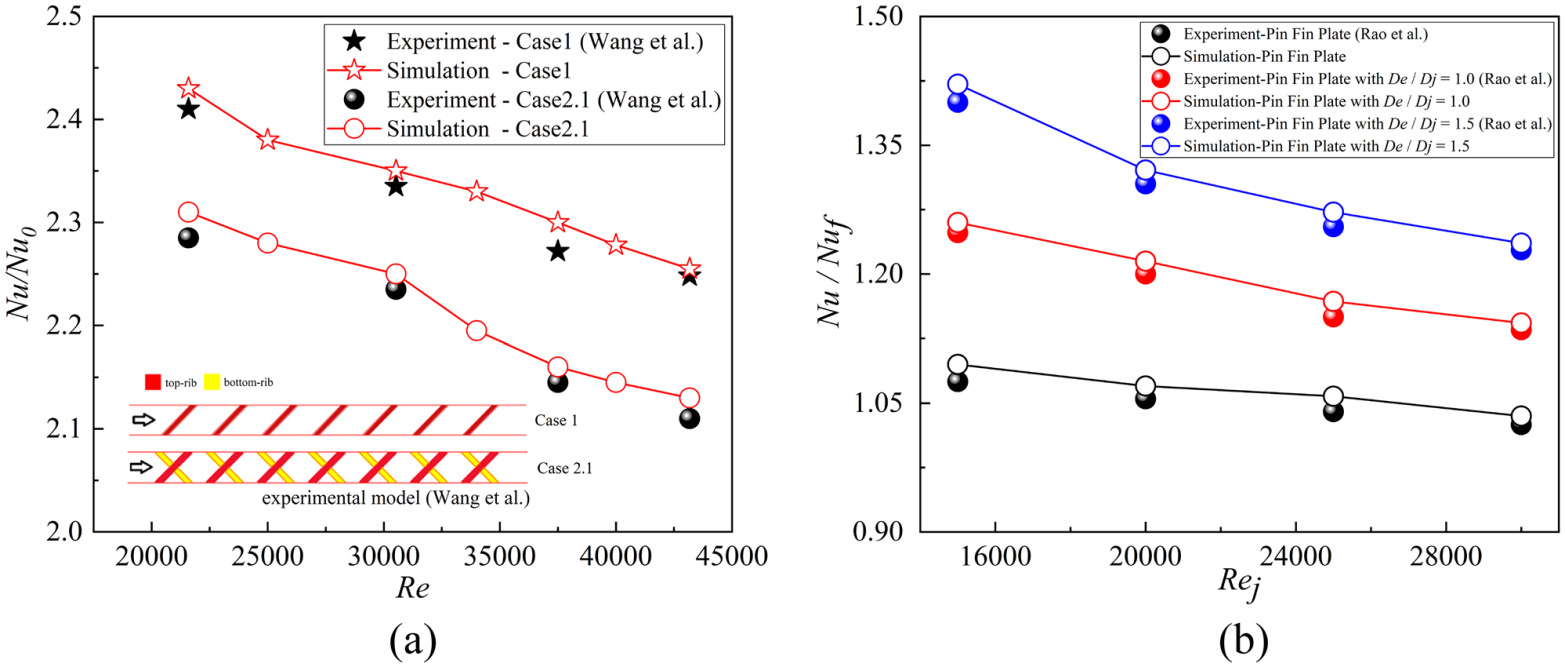
Numerical validation of internal cooling with the experimental results^36,37^. (**a**) Local heat transfer with ribs ^36^; (**b**) Multiple-jet impingement heat transfer with pin fins and effusion holes^37^.

To prove the reliability of the simulation further, the published experimental results were utilized to verify the grid computing and simulation methods, as shown in Figs. 10, 11. Firstly, the overall cooling effectiveness of Xie et al.^28^ was verified. The results show that the numerical simulation is consistent with the experimental results under different blowing ratios. The maximum error is 12% at *x*/*D* = 8. When *x*/*D* is greater than 20, the error is small. Figure 10b provides a schematic view of test Section^28^. Xie et al.^28^ filled the insulation material around the flat plate to reduce the effect of heat conduction and to match the boundary conditions of the simulation. Although the insulation material can play a certain role, heat conduction still exists compared with the adiabatic wall in the numerical simulation. The first half of the flat plate was heated by the channel; therefore, *ϕ* of the experiments was lower than that of the simulation results. Overall, considering that 100% adiabatic conditions could not be achieved in the experiments, the numerical simulation method used in this study could match the experimental results and had high reliability. This study also verified the accuracy of numerical simulation with internal cooling (ribs and impingement effusion), as shown in Fig. 11. Compared with the experimental results of Wang et al.^36^ and Rao et al.^37^, it could be seen that the numerical simulation accurately obtained the results of *Nu*. The simulation overestimated the internal heat transfer with a maximum error 2%. Compared with the results of overall cooling effectiveness, the numerical methods adopted in this study could predict the internal heat transfer more accurately. In general, the numerical methods with SST *k–ω* turbulence model in this study have been verified in detail.

**Figure 12:**
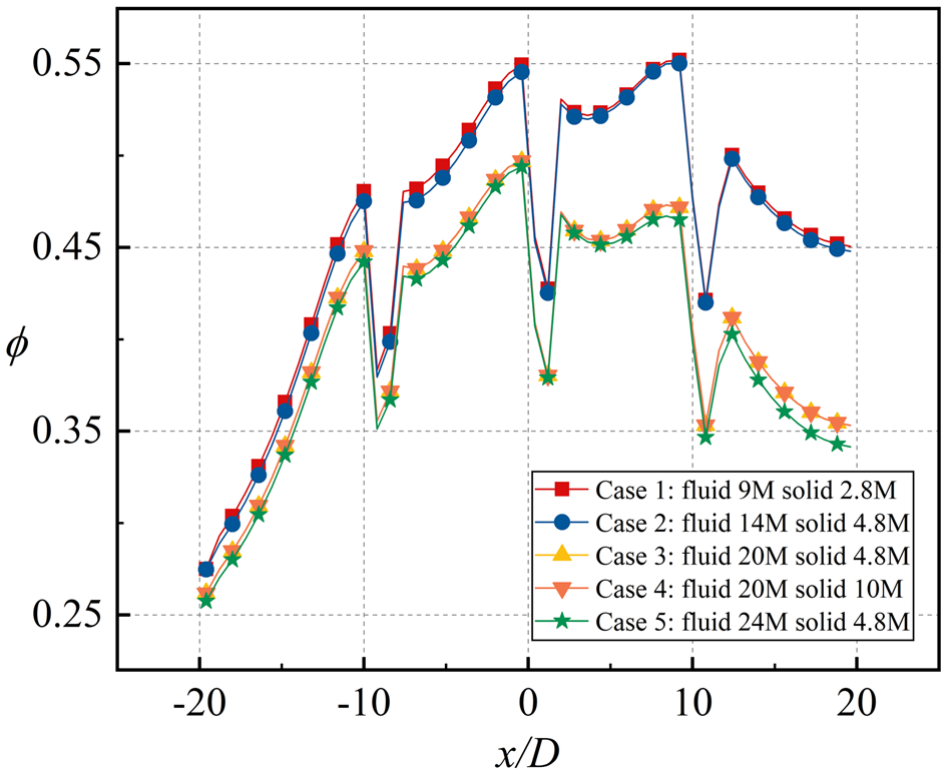
Grid number sensitivity.

To satisfy the requirements of the SST *k–ω* turbulence model (*y*^+^ = 1), the height of the first-layer grid was 0.008 mm. The prism was set at the couple wall to satisfy the requirements of the boundary layer grid. To eliminate the effects of the grid on the simulation, the grid sensitivity was verified, as shown in Fig. 12. The results for Cases 3 and 4 were similar. To save computing resources, the grids of the solid and fluid domains were set as 4.8 million and 20 million, respectively.

## Results and Discussion

This section presents the contours of overall cooling effectiveness firstly. Then the laterally averaged and area averaged results of adiabatic film cooling effectiveness, heat transfer coefficient ratio, and Biot number for different cases will be given to analyze the overall cooling effectiveness. And the effects of the internal cooling structures, film hole structures, and *Re*_*g*_ on the three dimensionless parameters are also described. Finally, the one-dimensional conjugate heat transfer model is verified.

### Overall cooling effectiveness

**Figure 13:**
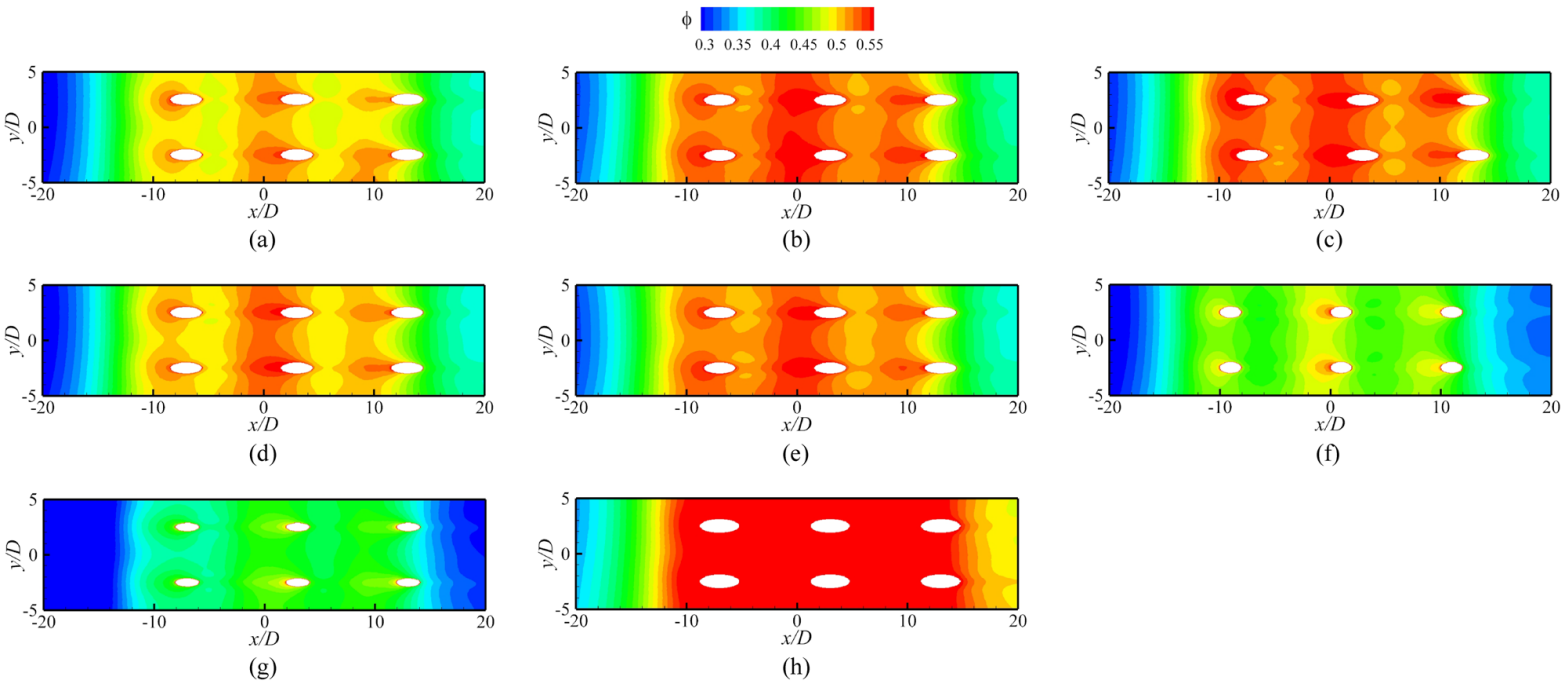
Contours of overall cooling effectiveness for film hole model. (**a**) Ref-case #1; (**b**) 45° rib; (**c**) 90° rib; (**d**) Dimple; (**e**) V rib; (**f**) *α* = 30°; (**g**) *D*_*film*_ = 0.75*D*; (**h**) *D*_*film*_ = 1.25*D*.

Figure 13 shows the overall cooling effectiveness of different internal cooling and film hole structures for the film hole model. The results indicate that the rib turbulator can improve the overall cooling effectiveness. The improvements of the 45° and 90° ribs are the most obvious, followed by that of the dimple structure. When the film hole inclination angle *α* is increased to 30°, *ϕ* decreases significantly. With the increase of *D*_*film*_, *ϕ* gradually increases.

**Figure 14:**
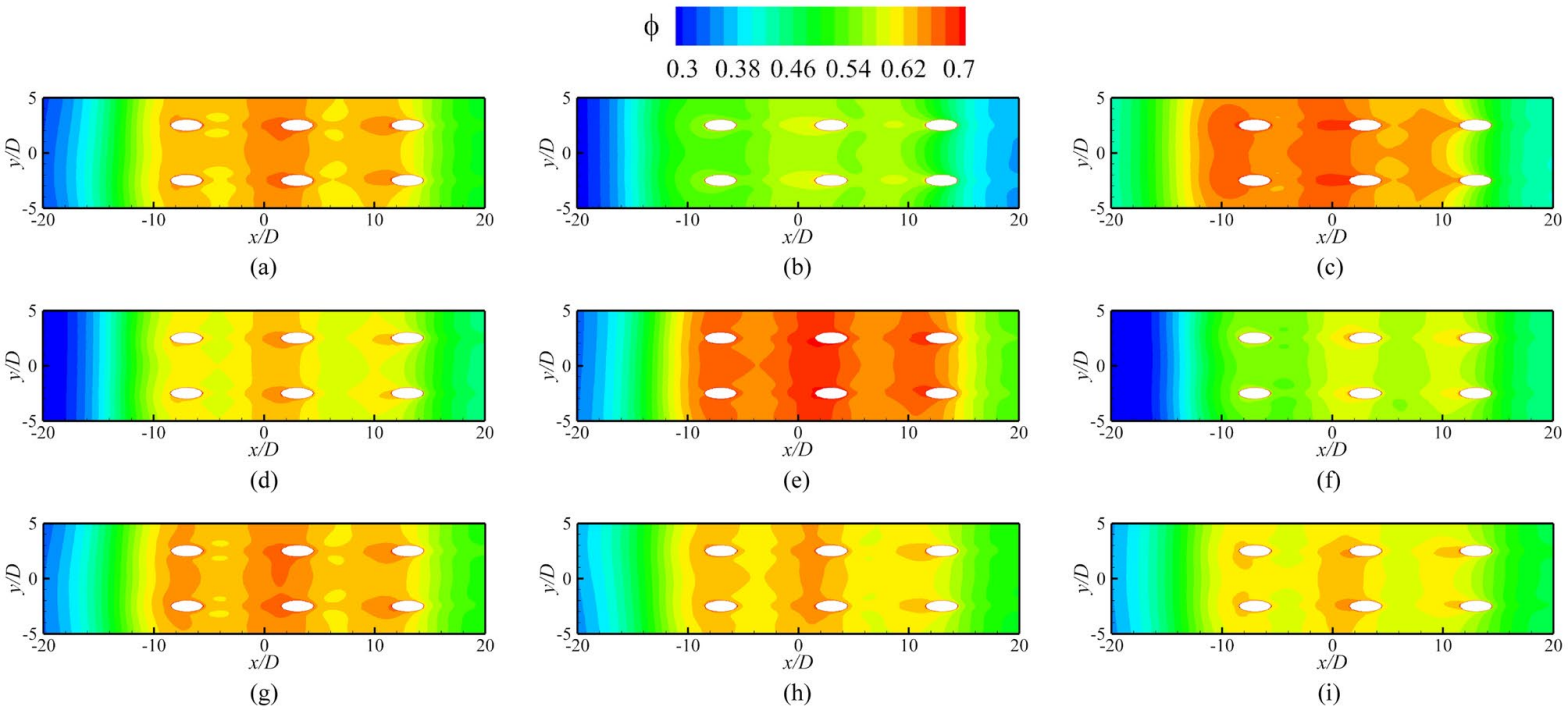
Contours of overall cooling effectiveness for impingement effusion model. (**a**) Ref-case #2; (**b**) Overlapped; (**c**) Span parallel; (**d**) Streamwise parallel; (**e**) *D*_*imp*_ = 1.5*D*; (**f**) *D*_*imp*_ = 2.5*D*; (**g**) *t*_*gap*_ = 5*D*; (**h**) *t*_*gap*_ = 10*D*; (**i**) *t*_*gap*_ = 15*D*.

**Figure 15:**
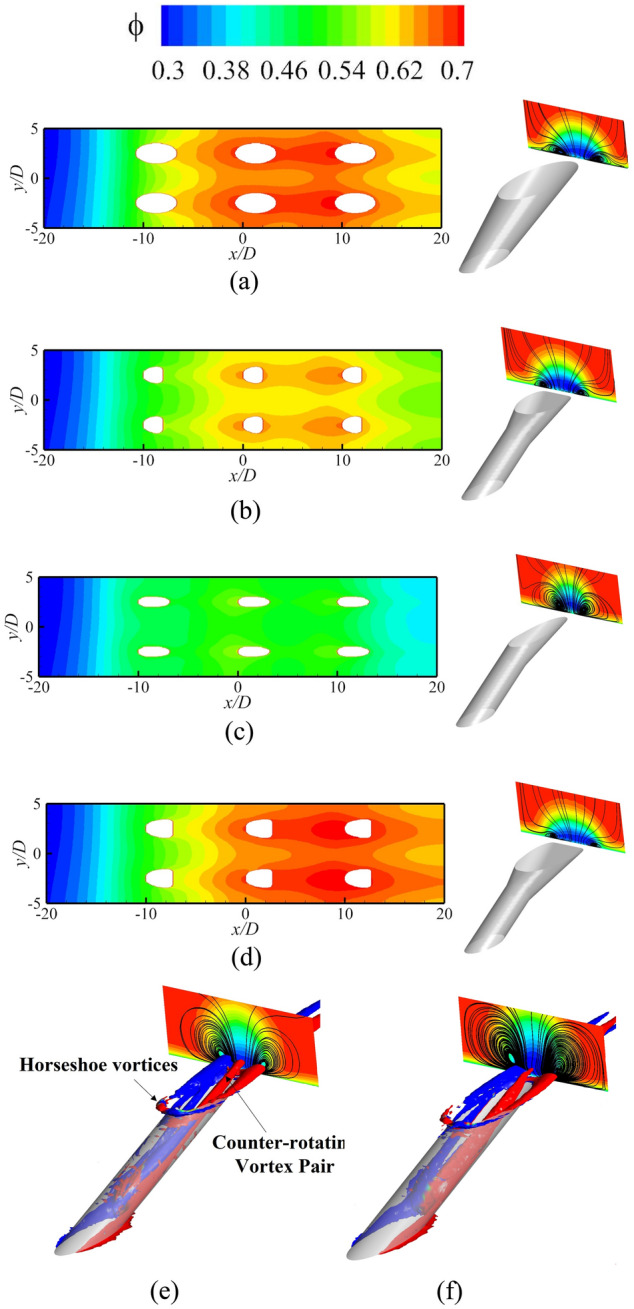
Contours of overall cooling effectiveness for different shaped hole structures. (**a**) Conical; (**b**) Fan-shaped; (**c**) Laidback; (**d**) Laidback fan-shaped; (**e**) Ref-case #1; (**f**) Smooth (*D*_*film*_ = 1.25*D*).

**Figure 16:**
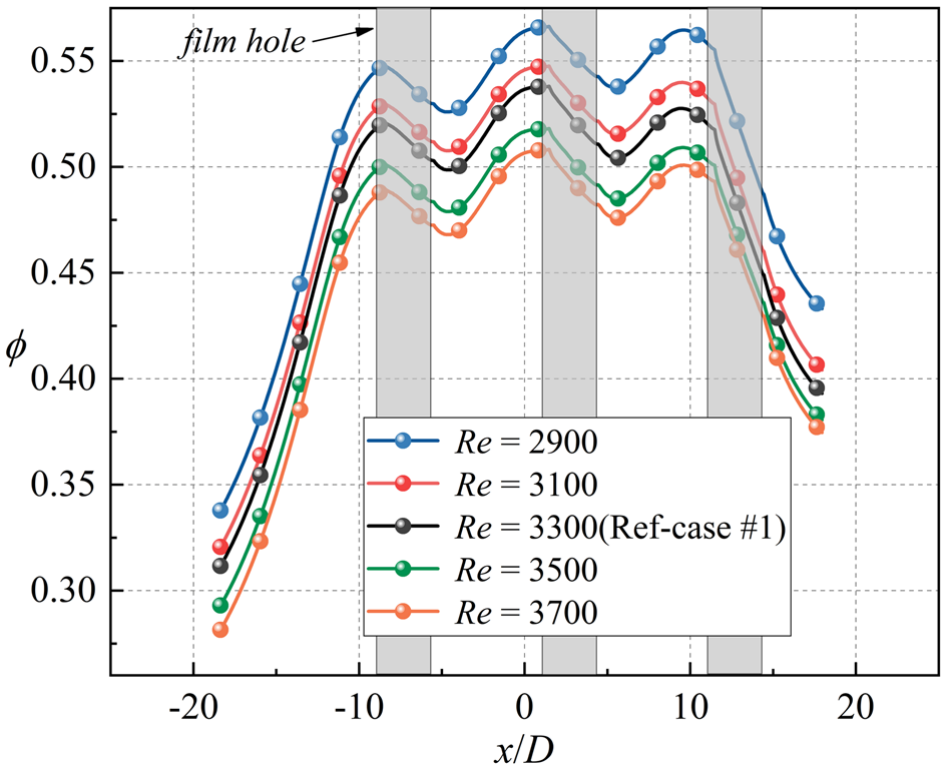
Laterally-averaged overall cooling effectiveness of film hole models with different Reynolds numbers.

Figure 14 presents *ϕ* of the impingement-effusion model with different cooling structures. Compared with the results in Fig. 13, the impingement flow can significantly improve *ϕ*. The distribution of impingement holes also influences the overall cooling effectiveness. In addition to the span parallel structure, the overlapped and streamwise parallel structures will weaken the overall cooling effectiveness compared with Ref-case #2. With the increase of impingement hole diameter *D*_*imp*_, the decrease in jet velocity weakens the heat transfer enhancement. Therefore, the overall cooling effectiveness also gradually decreases. The overall cooling effectiveness remains almost constant until the gap distance increases to 10*D*. The shaped-hole structures can also effectively improve *ϕ*, as evidenced by comparison of Fig. 13. The impingement effusion structure and shaped hole structures improve *ϕ* by increasing the internal heat transfer coefficient (*h*_*i*_) and adiabatic film cooling effectiveness (*η*), respectively. By comparing Fig. 15a–d with Fig. 15e, it can be seen that the shaped hole structure can weaken the Counter-rotating Vortex Pair (CVP), and this weakening effect is not due to the increase of the film hole outlet area. Figure 15f depicts the streamline of the CVP when *D*_*film*_ is 1.25*D*. The results demonstrate that the CVP is not weakened with the increase of *D*_*film*_. Figure 16 shows the laterally averaged overall cooling effectiveness of the film hole model under different *Re*_*g*_. With the increase of *Re*_*g*_, the enhancement of the heat transfer on the mainstream side reduces the overall cooling effectiveness.

### Effects of internal cooling structure

According to Eq. (17), the overall cooling effectiveness *ϕ* is related to the adiabatic film cooling effectiveness *η*, the heat transfer coefficient ratio *h*_*g*_/*h*_*i*_ and the Biot number *Bi*_*g*_. This section describes the effects of internal cooling structures, such as the gap distance, impingement hole diameter, rib turbulator and impingement hole distribution on these three dimensionless parameters.

**Figure 17:**
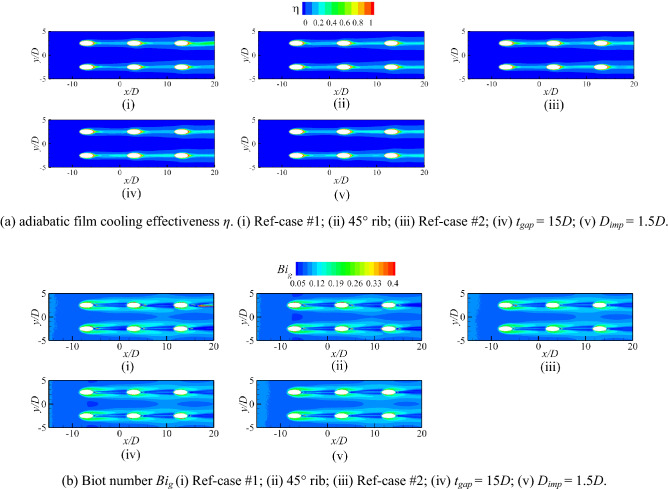
Effects of internal cooling structures on *η* and *Bi*_*g*_.

**Figure 18:**
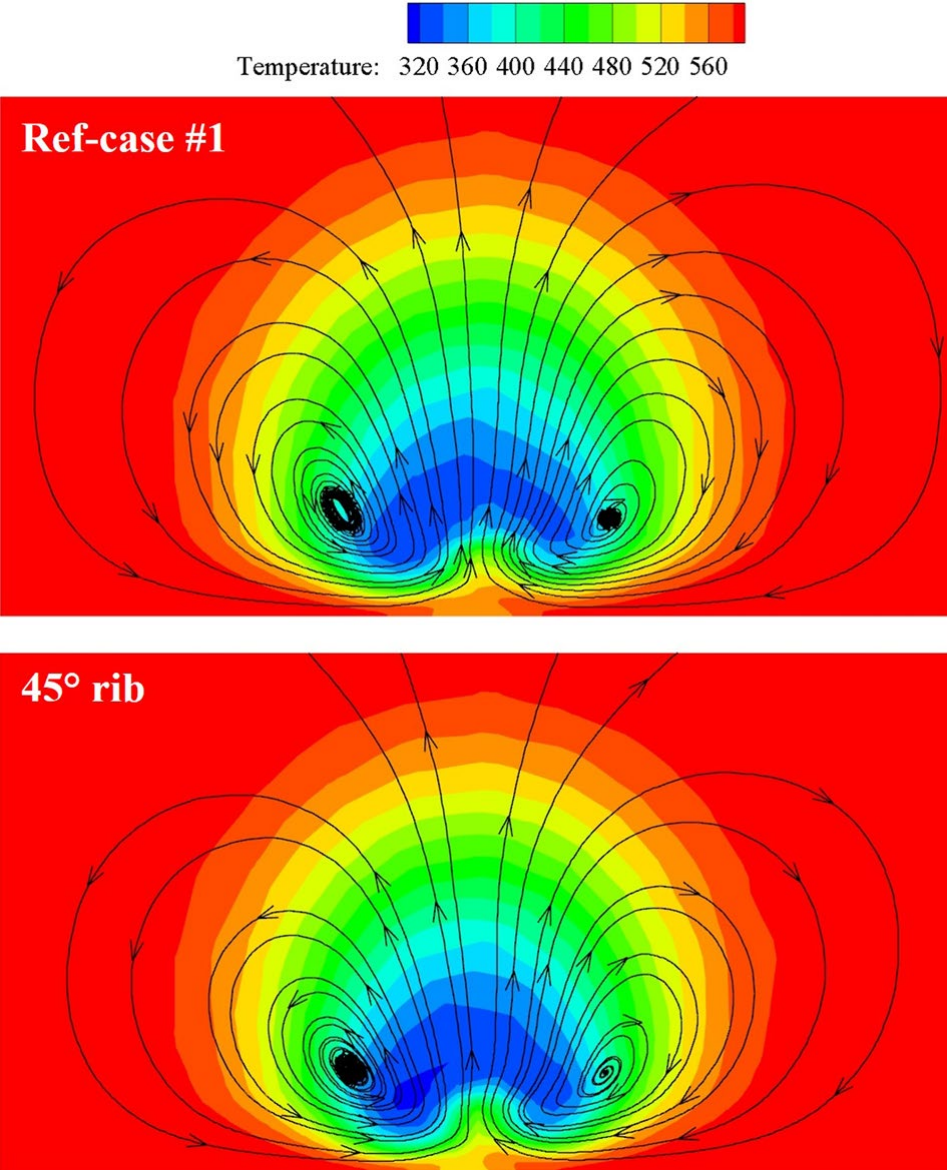
Temperature distribution and streamlines at slice *x*/*D* = 6.5.

Figure 17 shows the contours of *η* and *Bi*_*g*_. The results showed that the internal cooling structures hardly affected the distribution of *η*. Vast studies have proven that the interaction between the coolant and mainstream causes complex vortical structures, such as Windward Vortices (WV), Counter-rotating Vortex Pair (CVP), Horseshoe Vortices (HV). Among many vortical structures, the CVP is considered to be the dominant vortex that can influence film hole cooling^38^. Figure 18 presents the temperature distribution and streamlines of Ref-case #1 and the 45° rib case at the outlet of the film hole. The CVP is almost unaffected by the internal cooling structures; therefore, adiabatic film cooling effectiveness is almost unchanged. Although the internal cooling structure hardly affects the distribution of *η* (i.e., *T*_*aw*_), it affects the heat flux *q*_*g*_ and temperature *T*_*w*_ on the mainstream side. According to Eq. (6) and the definition of *Bi*_*g*_ (= *h*_*g*_*δ*/*k*), the thickness of the film hole plate *δ* is a small scale, which weakens the influence of the internal cooling structure on the external heat transfer coefficient *h*_*g*_. Therefore, the influence of the internal cooling structure on *Bi*_*g*_ is also small, as shown in Fig. 17b. In summary, the internal cooling structure has little effect on the distributions of *η* and *Bi*_*g*_. The key to improve the overall cooling effectiveness by optimizing the internal cooling structures is to improve the internal heat transfer coefficient *h*_*i*_.

**Figure 19:**
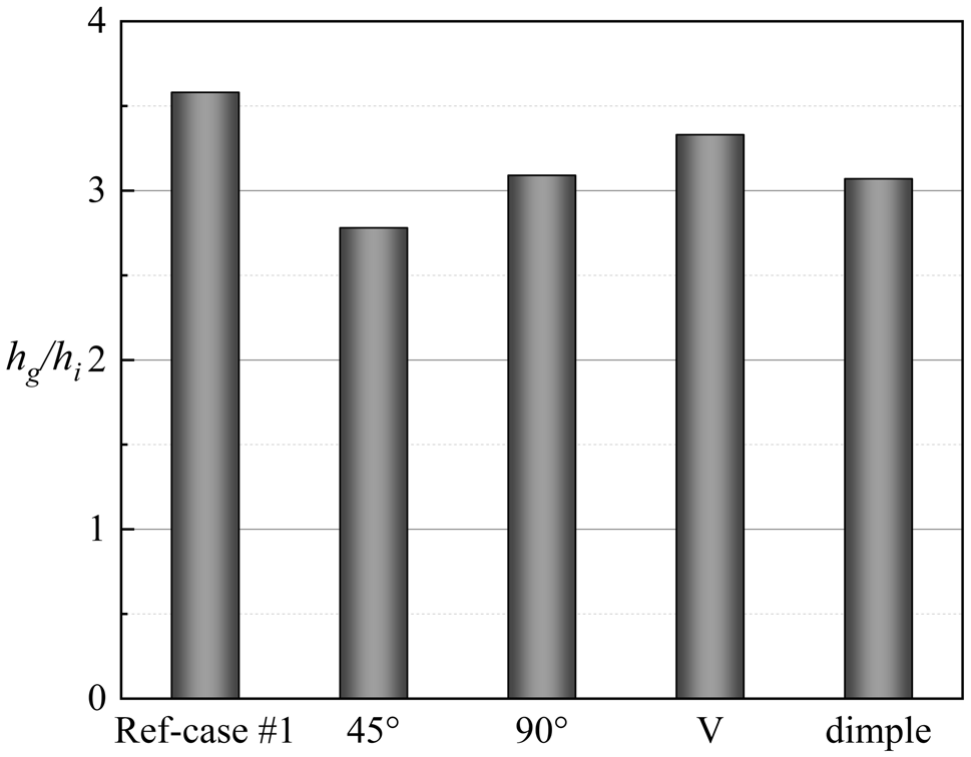
Area-averaged *h*_*g*_/*h*_*i*_ for film hole model with *α* = 20°.

**Figure 20:**
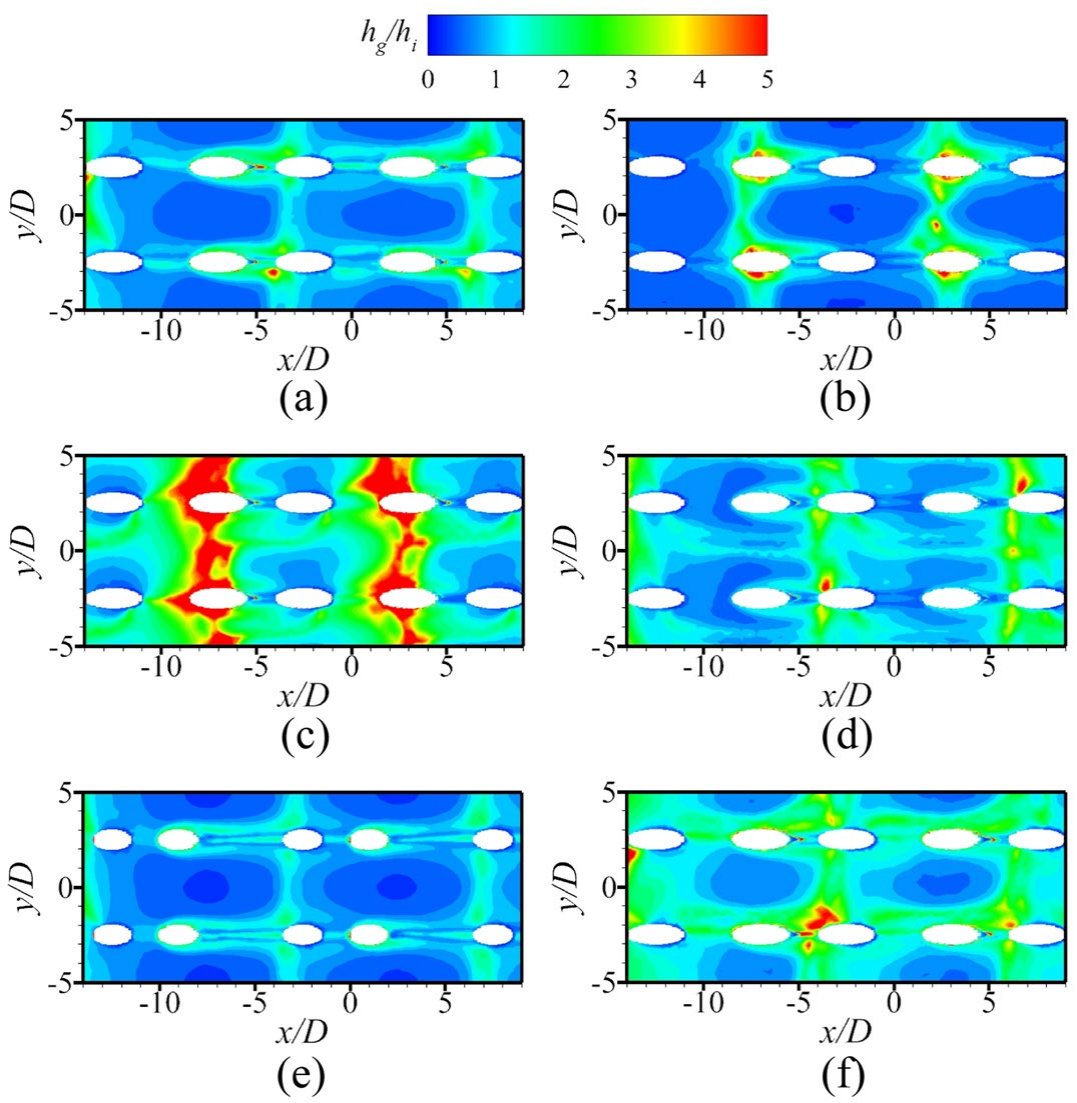
Contours of *h*_*g*_/*h*_*i*_ for impingement-effusion model (first two rows: different impingement hole distributions; last row: different impingement hole diameter *D*_*imp*_). (**a**) Ref-case #2; (**b**) Span parallel; (**c**) Overlapped; (**d**) Streamwise parallel; (**e**) *D*_*imp*_ = 1.5*D*; (f) *D*_*imp*_ = 2.5*D*.

The above discussions analyze the influence of the internal cooling structure on external film cooling (*η* and *Bi*_*g*_). According to Eq. (17), another dimensionless parameter that can determine the overall cooling effectiveness is the heat transfer coefficient ratio *h*_*g*_/*h*_*i*_. Figures 19, 20 presents the results of the heat transfer coefficient ratio *h*_*g*_/*h*_*i*_ for the different internal cooling structures. The rib turbulator reduces *h*_*g*_/*h*_*i*_, as shown in Fig. 19. Because rib turbulator hardly affects the *η* and *Bi*_*g*_, it increases the overall cooling effectiveness by reducing *h*_*g*_/*h*_*i*_.

**Figure 21:**
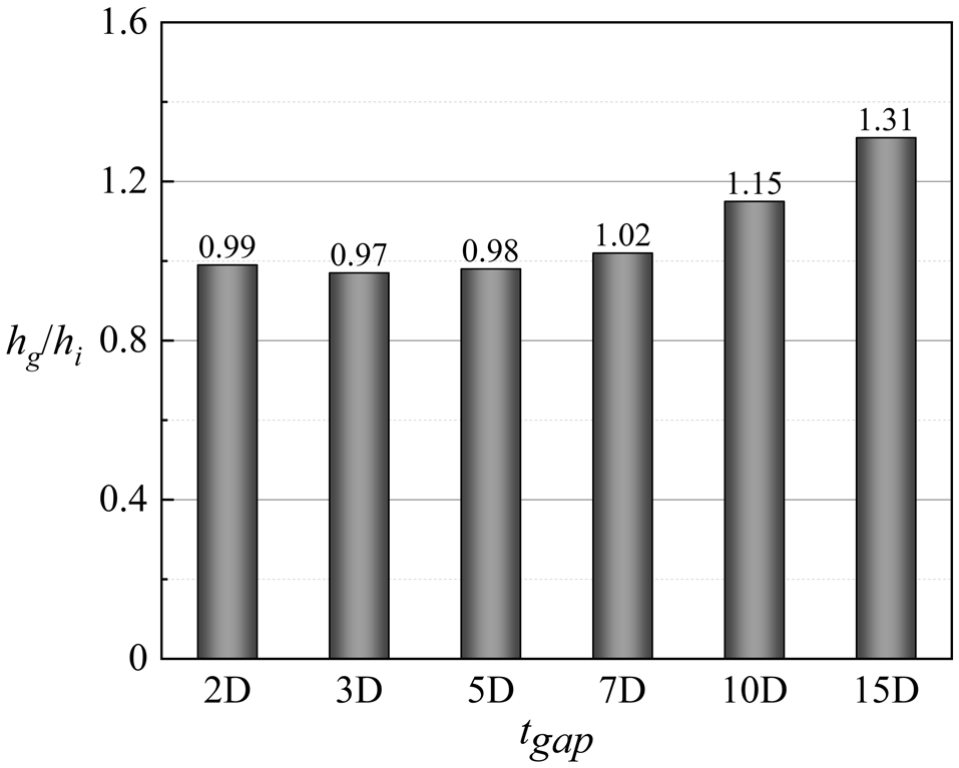
Area-averaged *h*_*g*_/*h*_*i*_ for impingement-effusion model with different gap distances.

According to the definition of *Bi*_*g*_ (= *h*_*g*_*δ*/*k*), *Bi*_*g*_ can represent the distribution of *h*_*g*_. A decrease in *h*_*g*_/*h*_*i*_ can improve *ϕ*, as shown in Eq. (17). Therefore, under the premise that the internal cooling structures have little effect on *Bi*_*g*_ and *η*, it is important to improve *ϕ* by enhancing *h*_*i*_ in regions where *Bi*_*g*_ is high. As shown in Fig. 17, the large *Bi*_*g*_ region is mainly near the outlet of the film hole. Figure 20 shows *h*_*g*_/*h*_*i*_ for cases with different impingement hole distributions and diameters *D*_*imp*_. The *h*_*g*_/*h*_*i*_ value of the span parallel layout is the smallest, followed by that of the staggered layout (Ref-case #2). The *h*_*g*_/*h*_*i*_ gradually decreases with increasing *D*_*imp*_; thus, *ϕ* also gradually decreases. As shown in Fig. 21, *h*_*g*_/*h*_*i*_ remains constant until the gap distance increases to 10*D*.

The optimized internal cooling structure can improve the overall cooling effectiveness by enhancing the internal heat transfer to reduce the heat transfer coefficient ratio *h*_*g*_/*h*_*i*_. The other two dimensionless parameters *η* and *Bi*_*g*_ are insensitive to the internal cooling structure.

### Effects of film hole structure

**Figure 22:**
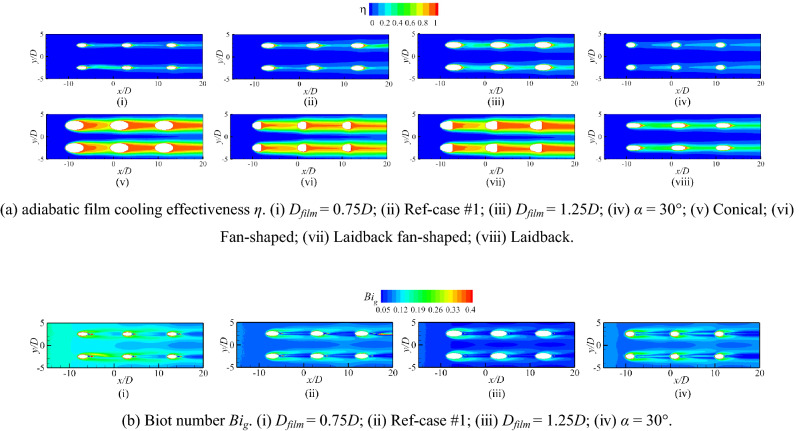
Effects of film hole structures on *η* and *Bi*_*g*_.

This section analyzes the effects of the shaped hole, film hole diameter *D*_*film*_, and film hole inclination angle *α* on the *η*, *h*_*g*_/*h*_*i*_, and *Bi*_*g*_. When the film hole structure changes, the coolant flow rate remains constant. With the increase of the film hole diameter *D*_*film*_, the velocity at the outlet of the film hole decreases, reducing the penetration of the coolant. Therefore, the adiabatic film cooling effectiveness gradually increases, as shown in Fig. 22i–iii. If the coolant flow rate remains unchanged, the outlet area of the film hole decreases when *α* = 30°; therefore, the velocity also increases. At the same time, *α* increases, which makes it easier for the coolant to be lifted off and reduces *η*. The interaction between the coolant and mainstream results in a CVP. Meanwhile, the hot mainstream follows the CVP to increase the temperature of the flow near the wall. According to the characteristics of the coolant jet, the key to improving *η* is to suppress the development of CVP^38^. Fan-shaped holes can cause less penetration and wider lateral-coverage owing to the diffused exit, as shown in Figs. 15, 22a. There is no diffused exit for the laidback hole, so it cannot result in wider lateral-coverage. However, a forward expansion angle can produce less penetration. Therefore, the shaped holes can effectively improve the adiabatic film cooling effectiveness, as shown in Fig. 22a.

**Figure 23:**
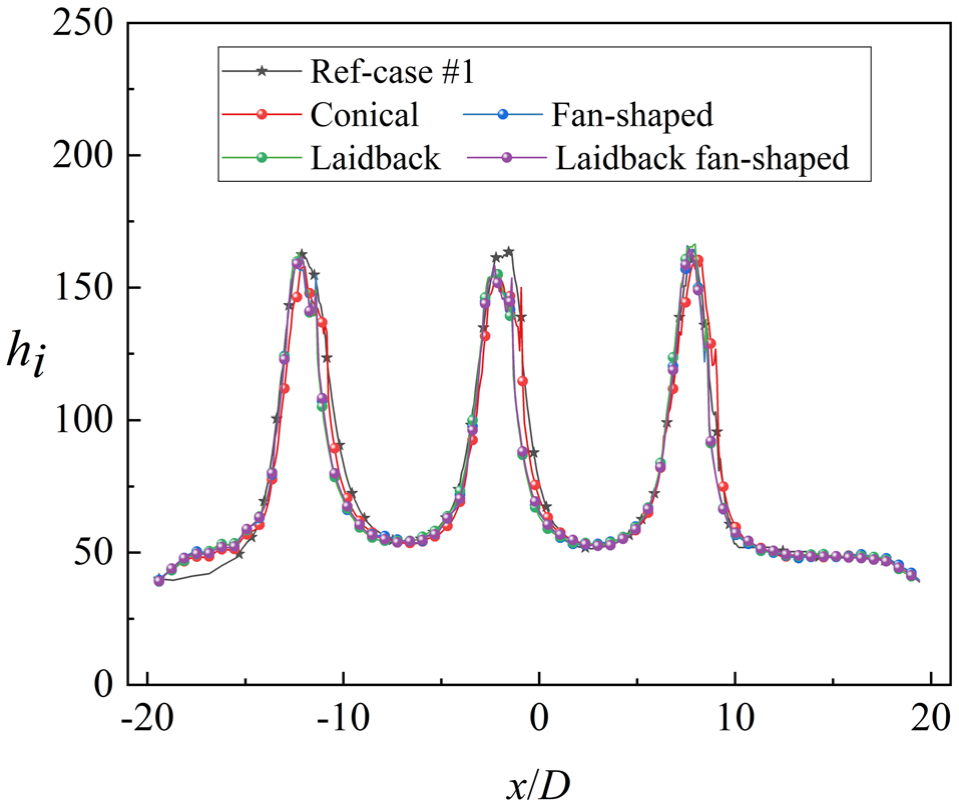
Laterally-averaged *h*_*i*_ for different shaped film hole structures.

**Figure 24:**
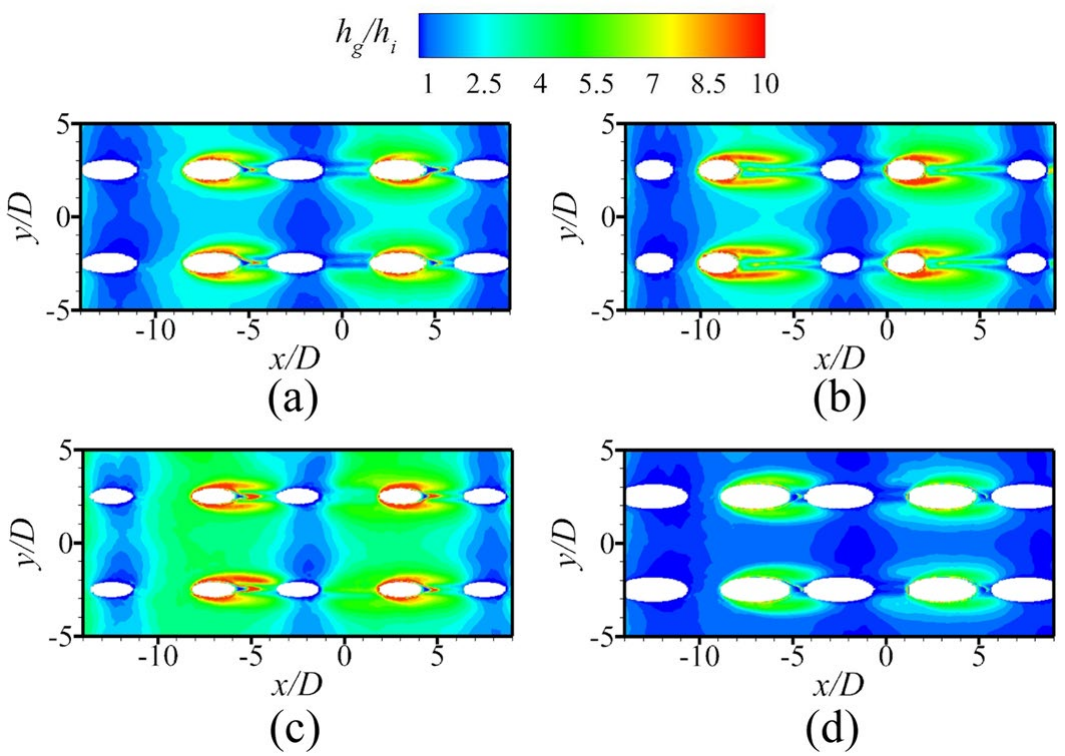
Contours of *h*_*g*_/*h*_*i*_ for film hole model. (**a**) Ref-case #1; (**b**) *D*_*film*_ = 1.0*D*, *α* = 30°; (**c**) *D*_*film*_ = 0.75*D*, *α* = 20°; (**d**) *D*_*film*_ = 1.25*D*, *α* = 20°.

Less penetration means lower Biot number *Bi*_*g*_^27^. With the increase of film hole diameter *D*_*film*_ and the decrease of film hole inclined angle *α*, the Biot number decreases. According to Fig. 23, the film hole structures have little effect on the internal heat transfer coefficient *h*_*i*_. Therefore, the influence of the film hole structure on *h*_*g*_/*h*_*i*_ is consistent with that on *Bi*_*g*_, as depicted in Figs. 22b, 24. In summary, the optimized film hole structure can improve *η* and reduce *Bi*_*g*_ and *h*_*g*_/*h*_*i*_*,* but has little effect on *h*_*i*_.

### Effects of aerodynamic parameters

**Figure 25:**
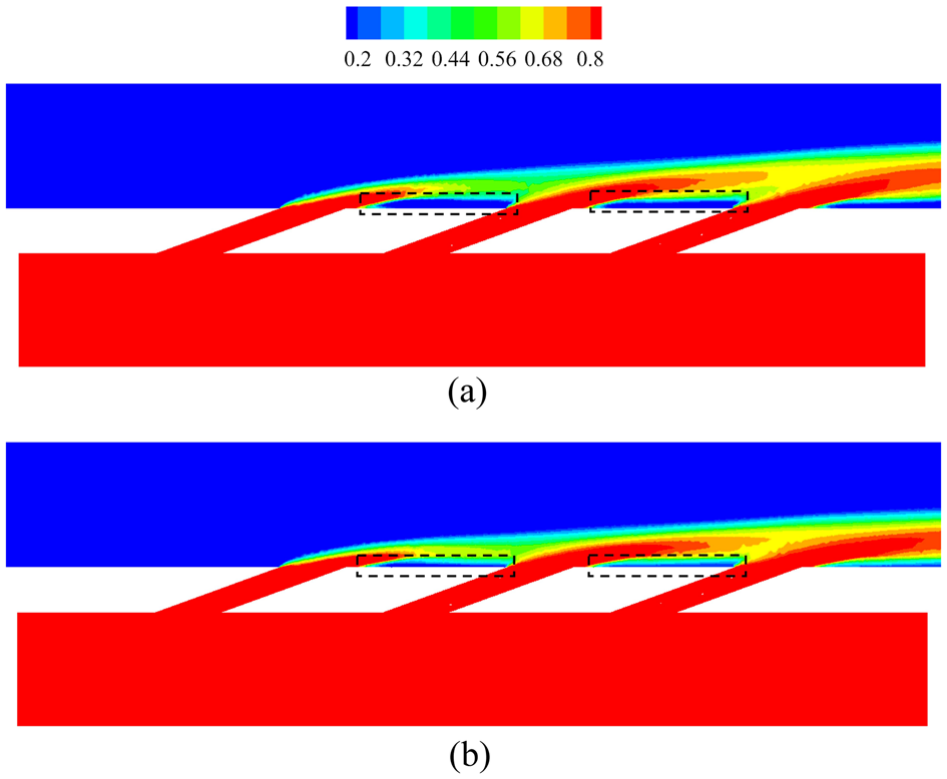
Distribution of dimensionless temperature* θ* at slice *y*/*D* = –2.5. (**a**) *Re* = 2900; (**b**) *Re* = 3700.

**Figure 26:**
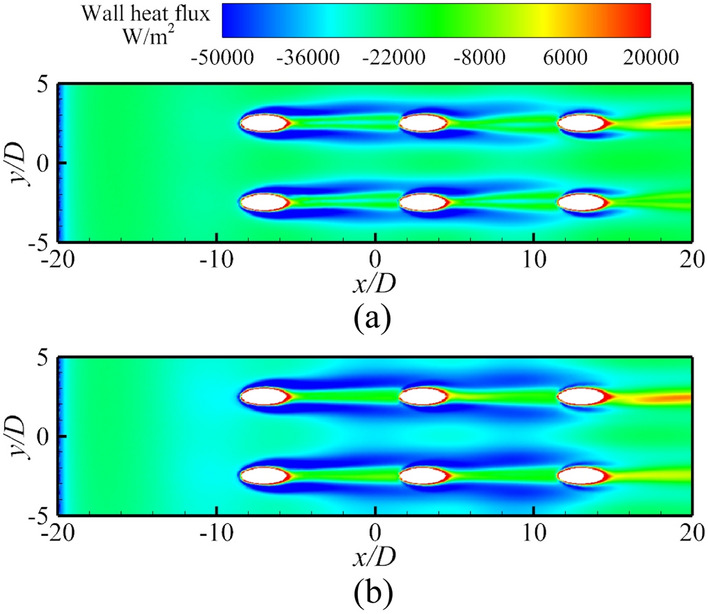
Contours of wall heat flux for Ref-case #1 with different *Re*_*g*_. (**a**) *Re* = 2900; (**b**) *Re* = 3700.

**Figure 27:**
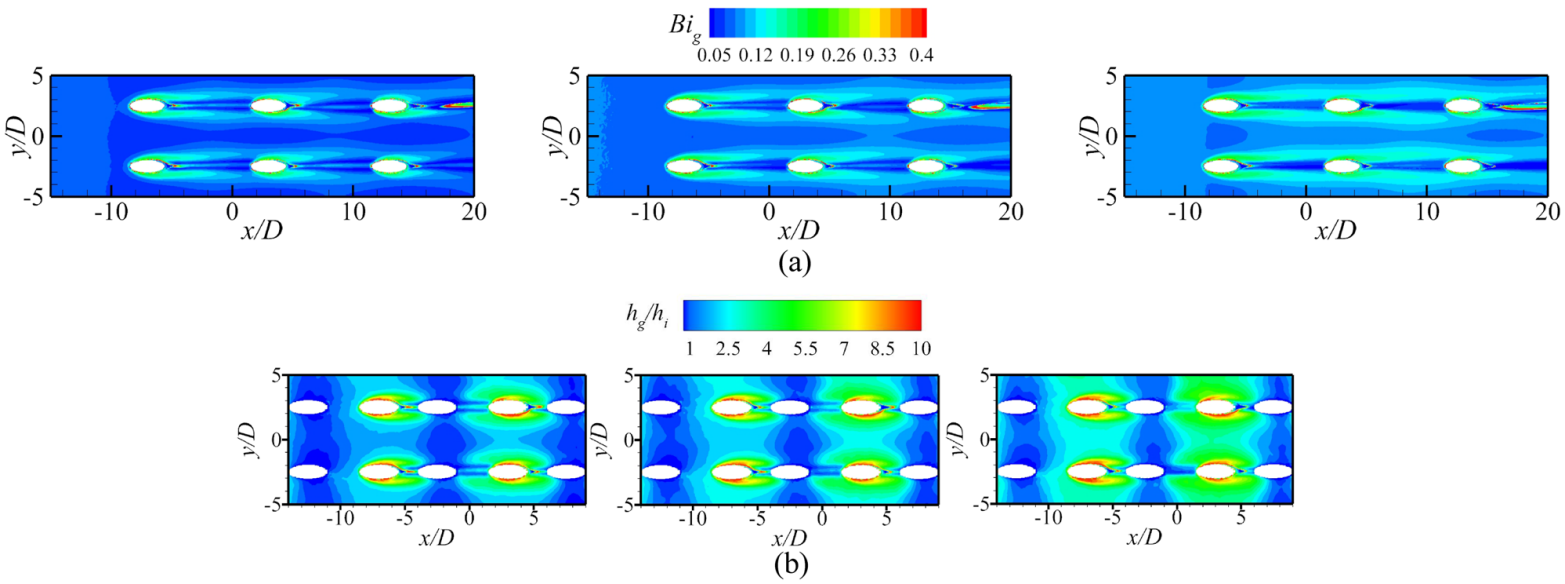
Contours of *Bi*_*g*_ and *h*_*g*_/*h*_*i*_ with different *Re*_*g*_ (left: *Re*_*g*_ = 2900; middle: *Re*_*g*_ = 3300 (Ref-case #1); right: *Re*_*g*_ = 3700). (**a**) Contours of *Bi*_*g*_; (**b**) Contours of *h*_*g*_*/h*_*i*_.

This section discusses the effects of *Re*_*g*_ on *η*, *h*_*g*_/*h*_*i*_, and *Bi*_*g*_. As shown in Fig. 25, the increase of *Re*_*g*_ can lead to less penetration, and thus, *η* will also be improved. However, the overall cooling effectiveness is related not only to *η*, but also to another two dimensionless parameters. The increase of *Re*_*g*_ increases the heat flux on the mainstream side, as shown in Fig. 26. The mainstream Reynolds number has little effect on the internal heat transfer coefficient *h*_*i*_. Therefore, with the increase of *Re*_*g*_, the *Bi*_*g*_ and *h*_*g*_/*h*_*i*_ also increase, as shown in Fig. 27. And this results in a decrease in overall cooling effectiveness.

### Validation of one-dimensional conjugate heat transfer model

According to Table 2, the overall cooling effectiveness is the most sensitive to the adiabatic film cooling effectiveness *η*, whereas the sensitivities to the Biot number and the heat transfer coefficient ratio are the same. However, according to Figs. 22, 24, the range of *h*_*g*_/*h*_*i*_ is larger than that of *Bi*_*g*_. Combined with Table 3, this section will verify the following two key conclusions: (1) with the increase of the heat transfer coefficient ratio *h*_*g*_/*h*_*i*_, the sensitivity of *ϕ* to *η* increases; and (2) the increase of adiabatic film cooling effectiveness *η* reduces the sensitivity of *ϕ* to *h*_*g*_/*h*_*i*_.

**Figure 28:**
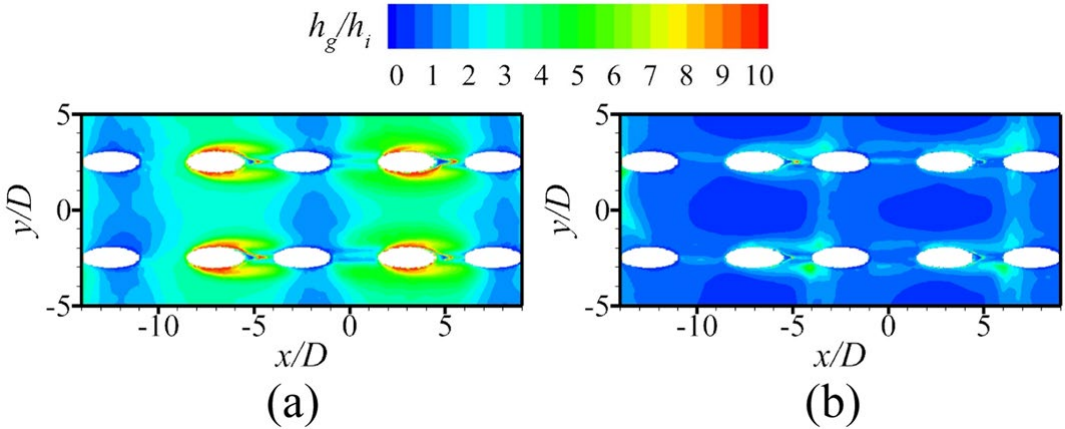
Contours of *h*_*g*_/*h*_*i*_ for different cases. (**a**) Ref-case #1 (smooth); (**b**) Ref-case #2 (smooth-imp).

**Figure 29:**
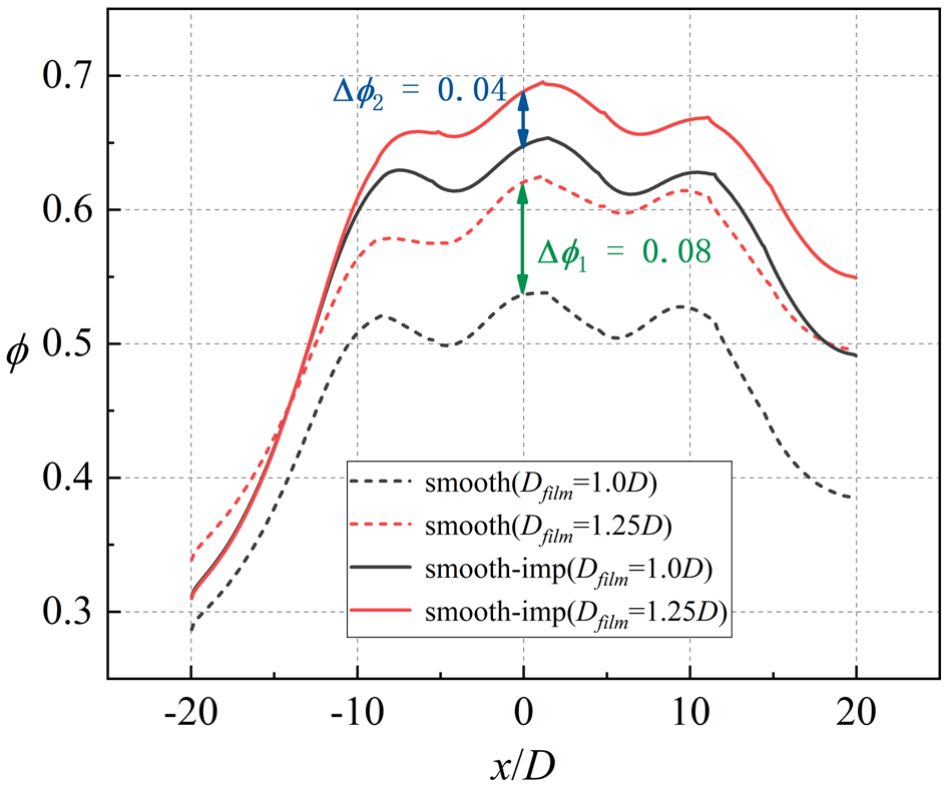
Laterally-averaged *ϕ* for different cases.

According to the above conclusions, the effects of the internal cooling structure on *Bi*_*g*_ and *η* can be ignored. Thus, Ref-case #1 and Ref-case #2 can be considered that only the heat transfer coefficient *h*_*g*_/*h*_*i*_ is different. The heat transfer coefficient ratio of Ref-case #2 is smaller than that of Ref-case #1 because of the impingement flow, as shown in Fig. 28. It can be observed from Fig. 22 that the increase of film hole diameter *D*_*film*_ can increase *η*. Considering that the effects of the internal cooling structure on *η* can be ignored, it is considered that the increase of *η* caused by the increase of *D*_*film*_ is roughly the same for Ref-cases #1 and #2. As shown in Fig. 29, when *D*_*film*_ increases from 1.0*D* to 1.25*D*, the overall cooling effectiveness (at *x*/*D* = 0) increases by 0.04 for Ref-case #2. For Ref-case #1, the overall cooling effectiveness increases by 0.08. When *η* increases by the same amount, the overall cooling effectiveness increases significantly for the case with a higher heat transfer coefficient ratio *h*_*g*_/*h*_*i*_ (Ref-case #1). In other words, when the overall cooling effectiveness increases by the same amount, *η* is required to increase less for the case with a higher heat transfer coefficient ratio (Ref-case #1). This confirms the first conclusion. With the increase of heat transfer coefficient ratio *h*_*g*_/*h*_*i*_, the sensitivity of overall cooling effectiveness to the adiabatic film cooling effectiveness *η* increases.

**Figure 30:**
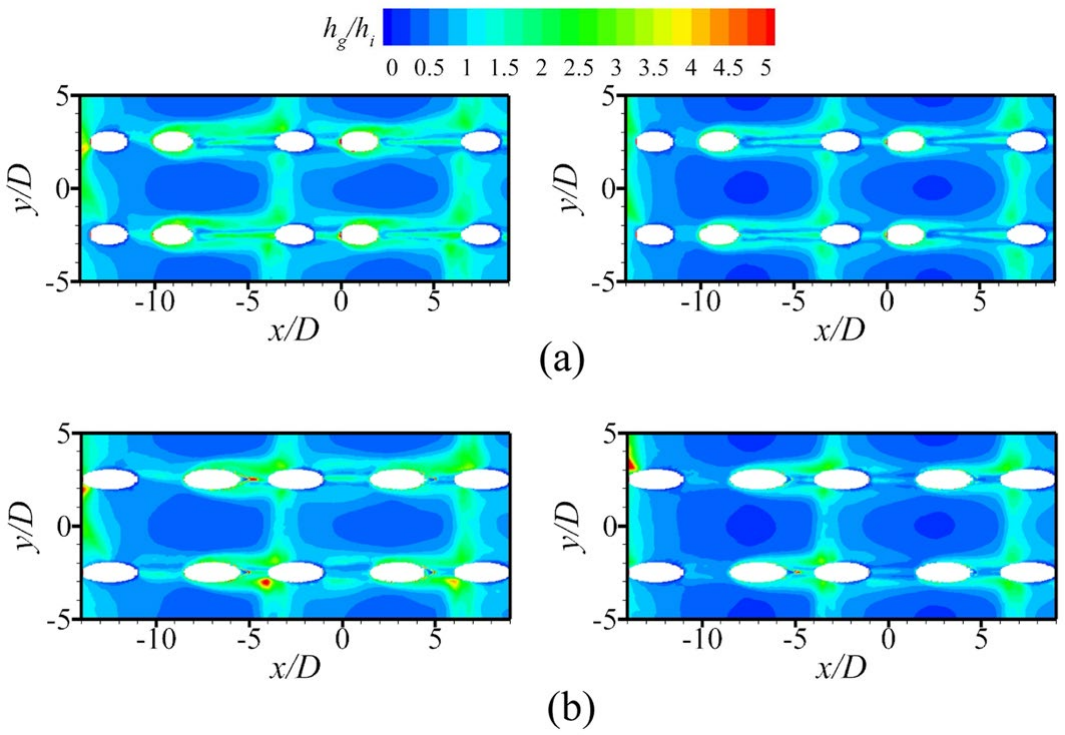
Contours of *h*_*g*_/*h*_*i*_ for impingement-effusion model (left: *D*_*imp*_ = 2D; right: *D*_*imp*_ = 1.5D). (**a**) *α* = 30°; (**b**) *α* = 20°.

**Figure 31:**
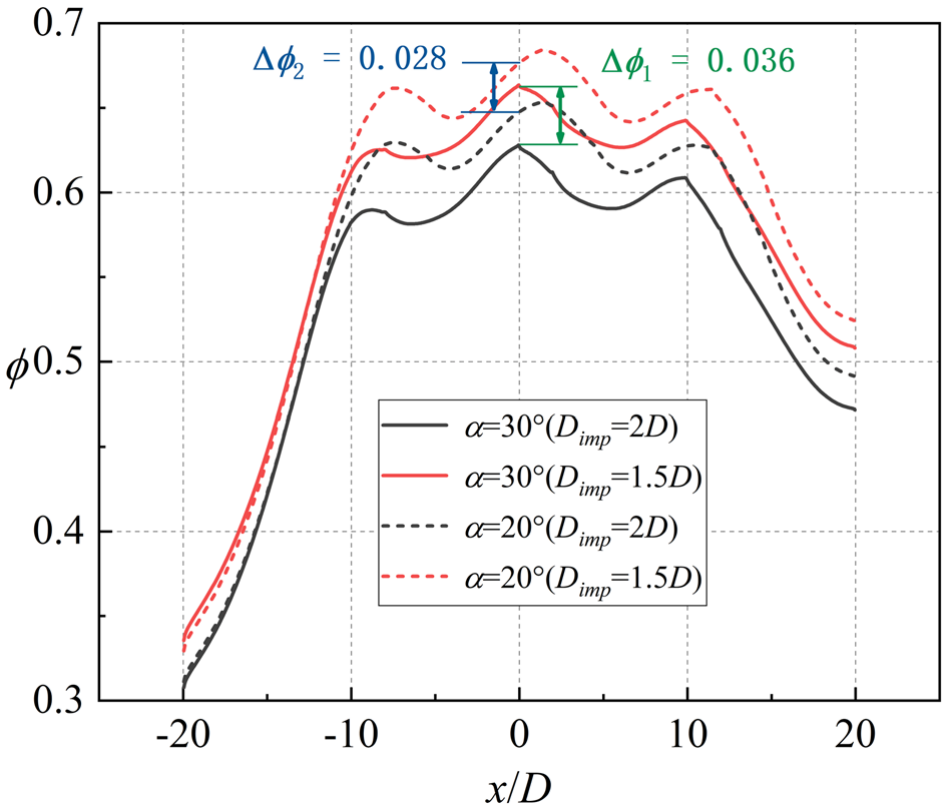
Laterally-averaged *ϕ* for impingement-effusion model.

As shown in Figs. 22, 24, *η* increases when *α* decreases from 30 to 20°. According to Fig. 17, *Bi*_*g*_ and *η* are almost unaffected by the impingement hole diameter *D*_*imp*_. As depicted in Fig. 30, the decrease of *h*_*g*_/*h*_*i*_ caused by the decrease of *D*_*imp*_ is almost identical for *α* = 30° and 20°. As illustrated in Fig. 31, as *D*_*imp*_ decreases from 2*D* to 1.5*D*, *ϕ* increases by 0.036 and 0.028, respectively. This indicates that when *h*_*g*_/*h*_*i*_ decreases by the same amount, the increase of overall cooling effectiveness decreases with the increase of *η*. In other words, the increase of adiabatic film cooling effectiveness *η* will reduce the sensitivity of overall cooling effectiveness to the heat transfer coefficient ratio *h*_*g*_/*h*_*i*_. The second conclusion is also confirmed.

The above discussion qualitatively verifies the conclusions regarding the sensitivity of *ϕ*. The sensitivity conclusions in Fig. 3 and Table 3 can provide a reference for the design of turbine blade cooling systems. To improve the practicability of Eq. (17), it is necessary to verify the accuracy of this model. Table 7 lists the area-averaged results of the three dimensionless parameters and the overall cooling effectiveness predicted by Eq. (17).

However, there must be deviations between the predictions obtained from Eq. (17) and the 3D numerical simulation. According to the definition of the Biot number (*B*_*i*_ = *hδ*/*λ*), this parameter characterizes the ratio of the thermal conduction resistance to the convective heat transfer resistance. Therefore, the larger the Biot number, the smaller the effects of thermal conduction. According to the definition of *Bi*_*c*_ (= *h*_*i*_*δ*/*λ*), the deviations of cases with higher *h*_*i*_ is small. According to Table 2, the adiabatic film cooling effectiveness accounts for the largest proportion in the overall cooling effectiveness compared with the other two dimensionless parameters. Therefore, the deviations caused by heat conduction will increase in the region far from the coolant coverage, and the prediction deviations will reduce for the cases with large adiabatic film cooling effectiveness. For Ref-case #1, there are no rib turbulator and impingement flow to enhance the internal heat transfer. Simultaneously, the coolant coverage is small because the exit is not diffused. Thus, the prediction deviation is too large to be accepted. However, the deviation is significantly reduced for cases with shaped holes or impingement flow, with a minimum error 4.3% for fan-shaped case. It indicates that the one-dimensional conjugate heat transfer model has an ideal applicability for these cases.

**Table 7:** Validation of one-dimensional conjugate heat transfer model.

Parameter	Ref-case #1	Laidback fan-shaped	Fan-shaped	Ref-case #2	*D*_*imp*_ = 1.5*D*	*t*_*gap*_ = 15*D*	Span parallel
*η*	0.119	0.495	0.468	0.113	0.115	0.113	0.1103
*Bi*_*g*_	0.106	0.094	0.115	0.106	0.105	0.114	0.099
*h*_*g*_/*h*_*i*_	3.636	3.25	3.897	0.886	0.666	1.01	0.875
*ϕ*_*pre*_	0.305	0.611	0.574	0.558	0.615	0.536	0.567
*ϕ*_*simulation*_	0.508	0.658	0.60	0.628	0.663	0.617	0.638
Deviation	39.9%	7.1%	4.3%	11.1%	7.2%	14.0%	11.1%

## Conclusions

In this study, the published one-dimensional conjugate heat transfer model was improved, and an equation relating *ϕ* to three dimensionless parameters (*η*, *Bi*_*g*_, and *h*_*g*_/*h*_*i*_) was obtained. The effects of these dimensionless parameters on the sensitivity of *ϕ* were also investigated using sensitivity charts. Simultaneously, flat film hole and impingement effusion model were established, which included different internal cooling structures and film hole structures. The effects of different internal cooling and film hole structures on three dimensionless parameters were studied by numerical simulation, and the accuracy of the conjugate heat transfer model was verified. The main conclusions can be summarized as follows.

1. The adiabatic film cooling effectiveness is positively correlated with the overall cooling effectiveness, while the Biot number and heat transfer coefficient ratio are negatively correlated with it. The overall cooling effectiveness is the most sensitive to adiabatic film cooling effectiveness among the considered parameters; therefore, increasing adiabatic film cooling effectiveness can improve the overall cooling effectiveness effectively.
2. The increase of heat transfer coefficient ratio can improve the sensitivity of overall cooling effectiveness to adiabatic film cooling effectiveness. The Biot number has little effect on the sensitivity of overall cooling effectiveness to the three dimensionless parameters. The increase of adiabatic film cooling effectiveness reduces the sensitivity of overall cooling effectiveness to heat transfer coefficient ratio.
3. The internal cooling structure has little effect on the adiabatic film cooling effectiveness and the Biot number. The impingent flow can significantly improve the overall cooling effectiveness by 20% compared to the smooth case without impingement flow.
4. The film hole structure has little effect on the internal heat transfer coefficient *h*_*i*_ but can change the distributions of *η* and *Bi*_*g*_. Compared with the other shaped hole structures, a laidback fan-shaped hole can most effectively improve *ϕ*. When the coolant flow rate remains constant, the increase of the film hole inclined angle can improve the adiabatic film cooling effectiveness, and reduce the heat transfer coefficient ratio and Biot number.
5. The increase of the mainstream Reynolds number *Re*_*g*_ will increase *h*_*g*_/*h*_*i*_ and *Bi*_*g*_.
6. The one-dimensional conjugate heat transfer model proposed in this study can accurately predict the overall cooling effectiveness for cases with impingement flow or shaped holes.

## Data Availability

The datasets used and/or analyzed during the current study available from the corresponding author on reasonable request.

## Abbreviations

*Bi*: Biot number (*Bi* = *h**δ*/*k*)
*D*: Diameter (mm)
*h*: Heat transfer coefficient (W/(m^2^·K))
*M*: Blowing ratio
*P*: Pressure (Pa)
*q*: Wall heat flux (W/m^2^)
*k*: Thermal conductivity (W/m·K)
*R**e*: Reynolds number (*Re* = *ρuD*/*μ*)
*t*: Thickness (mm)
*T*: Temperature (K)
*u*: Velocity (m/s)
*x*: Direction of the streamwise
*y*: Direction of the spanwise
*Y*: Dimensionless distance
*z*: Direction of the vertical

## Greek symbols

*α*: Film hole inclined angle (°)
*η*: Adiabatic film cooling effectiveness
*ϕ*: Overall cooling effectiveness
*ρ*: Density (kg/m^3^)
*θ*: Dimensionless temperature (*θ* = (600 − *T*)/(600 − 303))
*μ*: Dynamic viscosity (Pa s)
*χ*: Internal coolant warming factor

## Subscripts

*a**w*: Adiabatic wall
*c*: Coolant
*c**,exit*: Fluid at film hole exit
*c**,inlet*: Fluid at coolant inlet
*film*: Film
*gap*: Gap distance
*i*: Internal
*imp*: Impingement
*g*: Mainstream
*w*: Wall
